# Global, regional, and national burdens of endometriosis from 1990 to 2021: a trend analysis

**DOI:** 10.3389/fmed.2025.1562196

**Published:** 2025-08-14

**Authors:** Hailan Yan, Xiaoyan Li, Yi Dai, Jinghua Shi, Yushi Wu, Zhiyue Gu, Chenyu Zhang, Qiutong Li, Biyun Zhang, Shiqing Lyu, Jinhua Leng

**Affiliations:** ^1^Department of Obstetrics and Gynecology, Peking Union Medical College Hospital, Chinese Academy of Medical Sciences & Peking Union Medical College, Beijing, China; ^2^National Clinical Research Center for Obstetric & Gynecologic Diseases, Beijing, China

**Keywords:** endometriosis, disease burden, GBD 2021, incidence, DALYs

## Abstract

**Background:**

Endometriosis is a common, chronic, estrogen-dependent gynecological disorder that significantly affects patients’ quality of life. However, the disease burden and its trends may be undergoing significant changes, and comprehensive data on this issue are currently lacking. This study aimed to examine trends in the burden of endometriosis at the global, national, and regional levels.

**Methods:**

We collected detailed data on incidence numbers, disability-adjusted life years (DALYs), age-standardized incidence rate (ASIR), and age-standardized DALY rate (ASDR) for the period 1990–2021 from the GBD 2021 database. We calculated the estimated annual percentage changes (EAPCs) to quantify the temporal trends in the ASIR and ASDR of endometriosis and presented the global distribution of these trends.

**Results:**

The global incident cases of endometriosis were 3.45 million (95% uncertainty interval [UI] = 2.44 to 4.6), and DALYs were 2.05 million (95% UI = 1.20 to 3.13). The global ASIR decreased from 1990 to 2021 (EAPC = −1.01, 95% UI = −1.06 to −0.96), while the ASDR was similar (EAPC = −0.99, 95% UI = −1.04 to −0.95). The age groups with the highest global incidence and DALYs were 20–24 and 25–29 years. The highest 2021 ASIR and ASDR were both in Niger (77.33 per 100,000 individuals, 95% UI = 52.74 to 106.78; 61.45 per 100,000 individuals, 95% UI = 34.29 to 95.47) and in Oceania (77.71 per 100,000 individuals, 95% UI = 51.23 to 100.27; 45.24 per 100,000 individuals, 95% UI = 45.24 to 71.95). In 2021, the ASIR and ASDR were highest in the low sociodemographic index (SDI) region. We also found that the EAPC was negatively correlated with ASIR in 1990 (*ρ* = −0.55, *p* < 0.001) and positively correlated with Human Development Index (HDI) in 2021 (*ρ* = 0.556, *p* < 0.001).

**Conclusion:**

The global burden of endometriosis remains substantial and has continued to rise across most countries, territories, and regions since the 1990s, underscoring the urgent need for targeted public health strategies and advancements in diagnosis and treatment to address this pervasive condition.

## Introduction

Endometriosis is a chronic, common, and complex gynecological disorder defined as the presence of endometrial-like tissue outside the uterus (1). Endometriosis can cause pelvic pain and infertility, significantly impacting patients’ quality of life, such as daily activities, sexual health, and personal relationships (2, 3). In addition, endometriosis is associated with depression and fatigue, which can reduce work productivity and contribute to a considerable economic burden (1, 4).

Endometriosis is considered a global health concern, affecting approximately 5–10% of women of reproductive age (5), and has exhibited a global increase in incidence over the past decade (6). However, the disease burden and its trends may be undergoing significant changes, and comprehensive data on this issue are currently lacking.

Recently, the Global Burden of Diseases, Injuries, and Risk Factors Study (GBD) 2021 updated and systematically generated detailed estimates of global health and health loss, covering the distribution and burden of diseases across time, age, sex, location, and sociodemographic groups (7).

The present study is based on GBD 2021 estimates, encompassing annual case counts, incidence, and disability-adjusted life years (DALYs) of endometriosis. We aimed to investigate the global trends and distribution of endometriosis by performing a systematic analysis to yield new estimates of the incidence and DALYs of endometriosis from 1990 to 2021 at the global, regional, and national levels.

## Materials and methods

### Overview and case definition

In this study, we analyzed data on endometriosis from the Global Burden of Diseases, Injuries, and Risk Factors Study (GBD) 2021. For GBD 2021, endometriosis was confirmed by laparoscopy or laparotomy (7). The International Classification of Diseases, Tenth Edition (ICD-10) for endometriosis is N80-N80.9.^1^ To analyze global trends, we assessed the endometriosis trends according to the age stratification used in GBD 2021: 10–14, 15–19, 20–24, 25–29, 30–34, 35–39, 40–44, 45–49, 50–54, and >55 years old.

The GBD 2021 produced estimates of 371 diseases and injuries across 204 countries and territories, grouped into 21 regions and seven super-regions from 1990 to 2021 (7, 8). The GBD study used a standardized approach to estimate prevalence, incidence, mortality, years of life lost (YLLs), years lived with disability (YLDs), and DALYs, presenting these metrics as absolute numbers, age-standardized rates, and crude rates per 100,000 people (7). The eligibility criteria, literature search strategy, and data extraction methods for GBD 2021 have been thoroughly documented in previous studies (7). In the present study, we adopted this standardized GBD analytical framework to ensure consistency and comparability with previous GBD-based analyses. While this approach may limit methodological novelty, it allows for the generation of robust and comparable estimates over time and across regions, which is essential for global trend analysis.

The analysis and statistical code for estimating endometriosis are available on the GBD support website.^2^ A Bayesian meta-regression epidemiological tool, DisMod-MR 2.1, was used to generate incidence, prevalence, and remission estimates for endometriosis by age, sex, year, and location (7). In this study, we extracted numbers and rates on the incidence and DALYs of endometriosis at all age levels from GBD 2021 using the GBD Results Tool.^3^ The HDI (Human Development Index) data at country-level were sourced from the World Bank.^4^

The socio-demographic index (SDI) provides a summary measure of development that closely correlates with health outcomes. It is calculated as the geometric mean of three 0–1 scaled indicators: fertility rate among women under 25, average years of education for individuals aged 15 and older, and lag-distributed income per capita. For the GBD 2021 study, SDI values were scaled by a factor of 100 for reporting, where a score of 0 represents the lowest theoretical level of development related to health, and 100 represents the highest. A recent GBD 2021 capstone publication detailed the construction of SDI and categorized 204 countries into five quintiles—low, low-middle, middle, high-middle, and high—based on each country’s SDI value for 2021 (7).

### Statistical analysis

The estimated annual percentage changes (EAPCs) in the age-standardized incident rate (ASIR) and the age-standardized DALY rate (ASDR) were calculated to quantify the incidence trends of endometriosis. ASIR represents the number of cases per 100,000 individuals, while ASDR reflects years lived with disability per 100,000 individuals. We calculated age-standardized rates (ASR) per 100,000 individuals according to the following formula:


$$\mathrm{ASR}=\frac{\sum_{i=1}^{A}a_{i}w_{i}}{\sum_{i=1}^{A}w_{i}}\times 100,000$$

In the equation, i represents the i-th age group and $w_{i}$ is the population count (or weight) in the same age group i within the GBD 2021 global standard population, followed by dividing by the sum of the weights in the standard population.

EAPC is a comprehensive and commonly used measure for assessing ASR trends over a defined period. The calculation involves the natural logarithm of the weighted linear regression line fitted to ASR values, defined by γ=α+βx+ε, where x represents the calendar year. EAPC is derived using the formula, with a 95% confidence interval (CI) obtained from a linear regression model.


$$\mathrm{EAP}C_{\text{with}\;95\%\mathrm{CI}}=100\times (e^{\beta }-1)$$

If the estimated EAPC and the lower limit of its 95% CI are both greater than 0, ASR is considered to have an upward trend; if both the estimated EAPC and the upper limit of the 95% CI are less than 0, ASR is deemed to be decreasing. In cases where neither condition is met, ASR is regarded as stable.

To further investigate the factors affecting EAPC, we examined its correlations with the 1990 ASR and the 2021 HDI at the country level using Pearson’s correlation analysis. The 1990 ASR of endometriosis reflects the baseline disease prevalence, while the 2021 HDI serves as a proxy for each country’s healthcare quality and accessibility. The data can be accessed via the following link: https://github.com/YANN-GYN/GBD2021.git.

All analyses were performed using the R software (version 4.4.1)^5^. A *p*-value *of* < 0.05 was considered statistically significant.

The Institutional Review Board of Peking Union Medical College Hospital determined that the study did not need approval because it used publicly available data. This study complied with the Guidelines for Accurate and Transparent Health Estimates Reporting (GATHER) recommendations (9).

## Results

### Global endometriosis burden

**Figure 1:**
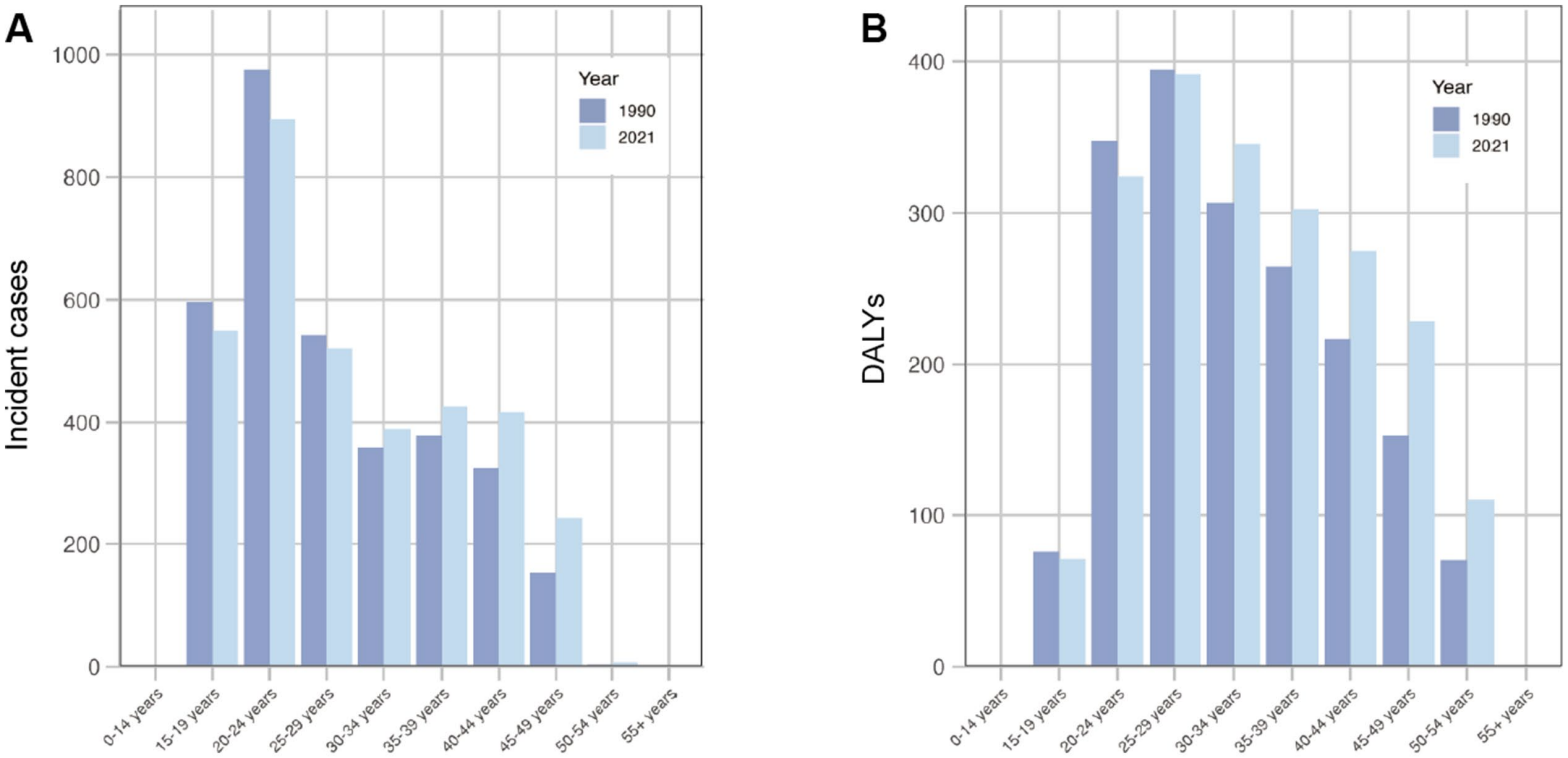
Global incident cases **(A)** and DALYs **(B)** of endometriosis by age group in 2019 and 2021.

Globally, the incident cases and DALYs of endometriosis increased from 1990 to 2021, reaching 3.45million (95% uncertainty interval [UI] = 2.44 to 4.6) and 2.05 million (95%UI = 1.20 to 3.13), respectively, in 2021 (Tables 1, 2). Compared to 1990, incident cases and DALYs had increased by 3.51 and 12.03%, respectively. In 1990 and 2021, the age groups with the highest incidence and DALYs of endometriosis were 20–24 and 25–29 years (Figure 1). For age groups over 30 years, incidence and DALYs in 2021 are generally higher than in 1990, showing a clear upward trend.

**Table 1:** Incident cases and ASIR of endometriosis in 1990 and 2021, and their temporal trends from 1990 to 2021.

Characteristics	1990		2021		1990–2021
	Cases No. × 10^3^ (95% UI)	ASIR per 100,000 No. (95% UI)	Case No. × 10^3^ (95%UI)	ASIR per 100,000 No. (95%UI)	EAPC No. (95% UI)
Global	3330.20 (2308.56–4506.99)	59.07 (41.2–79.24)	3447.13 (2436.26–4611.5)	43.64 (30.84–58.91)	−1.01 (−1.05 – −0.96)
Socio-demographic index					
High SDI	437.91 (305.8–594.89)	47.28 (33.03–64.08)	358.02 (260.64–467.06)	36.44 (26.19–47.95)	−0.98 (−1.05 – −0.91)
High-middle SDI	593.61 (412.14–799.09)	51.15 (35.67–69.44)	488.88 (353.41–647.89)	40 (28.64–53.11)	−0.78 (−0.87 – −0.69)
Middle SDI	1049.82 (715.02–1440.14)	55.11 (37.99–74.24)	1008.05 (709.01–1348.86)	40.51 (28.57–54.64)	−1.02 (−1.09 – −0.95)
Low-middle SDI	863.04 (596.98–1197.11)	74.09 (52.03–99.24)	981.57 (680.61–1336.89)	46.88 (32.76–63.34)	−1.49 (−1.51 – −1.46)
Low SDI	383.15 (264.54–531.72)	81.12 (57.08–109)	607.88 (420.34–848.82)	52.42 (36.38–71.48)	−1.41 (−1.45 – −1.36)
Region					
Central Asia	39.43 (26.98–54.98)	55.88 (38.92–75.45)	42.3 (29.47–56.25)	44.06 (30.63–59.03)	−0.45 (−0.62 – − 0.28)
Central Europe	50.95 (35.82–68.9)	41.65 (29.29–56.16)	36.13 (25.63–48.84)	37.17 (26.17–50.19)	−0.28 (−0.41 – −0.15)
Eastern Europe	148.32 (102.89–200.45)	68.05 (48.23–91.62)	117.89 (83.53–157.03)	66.2 (46.31–89.16)	0.31 (0.14–0.47)
Australasia	10.75 (7.5–14.68)	50.01 (34.79–68.28)	12.02 (8.3–15.98)	43.73 (30.33–58.89)	−0.27 (−0.34 – −0.19)
High-income Asia Pacific	110.47 (75.47–148.17)	59.12 (40.35–79.33)	77.24 (54.7–103.18)	50.72 (35.61–66.61)	−0.57 (−0.67 – −0.47)
High-income North America	133.51 (88.39–188.23)	43.32 (29.02–61.42)	91.19 (65.28–118.65)	26.9 (19.19–35.32)	−2.08 (−2.25 – −1.91)
Western Europe	140.95 (96.92–191.93)	36.83 (25.45–50.37)	126.35 (89.57–167.31)	35.55 (24.8–47.97)	0.01 (−0.03–0.06)
Andean Latin America	20.89 (14.23–29.33)	53.09 (36.87–71.63)	25.04 (17.3–34.48)	35.21 (24.41–48.43)	−1.26 (−1.32 – −1.19)
Caribbean	18.43 (12.71–25.93)	48.07 (33.32–65.47)	17.09 (11.78–23.45)	35.5 (24.49–48.73)	−0.95 (−0.97 – −0.93)
Central Latin America	88.22 (60.23–123.99)	50.83 (35.12–68.58)	89.76 (62.48–121.7)	33.29 (23.11–45.12)	−1.33 (−1.41 – −1.26)
Southern Latin America	21.13 (14.61–28.32)	42.57 (29.37–57.19)	24.59 (17.38–32.14)	35.09 (24.8–45.9)	−0.49 (−0.58 – −0.4)
Tropical Latin America	82.43 (55.41–114.84)	50.51 (34.43–69.81)	90.68 (63.37–120.41)	38.18 (26.53–51.36)	−1.23 (−1.38 – −1.07)
North Africa and the Middle East	259.96 (178.55–368.64)	75.18 (52.54–102.71)	341.35 (238.62–462.33)	51.09 (35.75–69.4)	−1.26 (−1.33 – −1.19)
Southeast Asia	341.47 (236.6–465.53)	69.16 (48.61–92.89)	386.14 (271.86–518.37)	51.79 (36.38–69.66)	−0.89 (−0.91 – −0.86)
South Asia	801.05 (554.34–1098.56)	71.91 (50.26–96.78)	936.79 (643.28–1268.52)	45.25 (31.32–61.09)	−1.51 (−1.52 – −1.5)
East Asia	704.5 (477.75–971.74)	49.73 (34.25–68.58)	444.7 (320.3–592.88)	32.04 (22.94–42.71)	−1.51 (−1.66 – −1.35)
Oceania	5.88 (4.07–8.08)	88.29 (62.08–118.63)	10.52 (7.36–14.57)	72.71 (51.23–100.27)	−0.61 (−0.63 – −0.59)
Central Sub-Saharan Africa	41.89 (28.62–58.53)	79.74 (55.41–107.44)	68.81 (46.62–96.83)	49.6 (33.83–67.94)	−1.45 (−1.56 – −1.34)
Eastern Sub-Saharan Africa	130.26 (89.67–181.63)	72.4 (49.97–98.25)	195.55 (135.17–273.39)	43.63 (29.96–59.93)	−1.62 (−1.67 – −1.58)
Southern Sub-Saharan Africa	33.5 (22.92–46.07)	60.54 (41.71–81.39)	40.51 (27.56–55.22)	46.23 (31.8–62.96)	−0.87 (−0.88 – −0.85)
Western Sub-Saharan Africa	146.21 (100.51–203.8)	79.05 (54.47–106.84)	272.48 (186.81–380.69)	55.16 (37.95–74.68)	−1.14 (−1.19 – −1.09)

ASIR, age standardized incidence rate; CI, confidence interval; EAPC, estimated annual percentage change; UI, uncertainty interval.

**Table 2:** DALYs and ASDR of endometriosis in 1990 and 2021, and their temporal trends from 1990 to 2021.

Characteristics	1990		2021		1990–2021
	Cases No. × 10^3^ (95%UI)	ASDR per 100,000 No. (95%UI)	Cases No. × 10^3^ (95%UI)	ASDR per 100,000 No. (95%UI)	EAPC No. (95% UI)
Global	1829.34 (1033.45–2864.71)	34.35 (19.58–53.96)	2049.47 (1195.2–3133.97)	25.37 (14.78–38.81)	−0.99 (−1.04 to −0.95)
Socio-demographic index					
High SDI	257.03 (147.4–414.12)	26.94 (15.43–43.11)	229.43 (137.2–353.69)	21.24 (12.8–32.29)	−0.91 (−1 to −0.82)
High-middle SDI	336.14 (190.14–528.33)	29.56 (16.82–46.63)	315.06 (187.39–494.01)	23.05 (13.73–35.24)	−0.75 (−0.83 to −0.66)
Middle SDI	545.39 (307.24–849.54)	31.19 (17.81–48.87)	604.49 (355.9–930.57)	23.12 (13.59–35.57)	−0.98 (−1.05 to −0.91)
Low-middle SDI	477.78 (274.69–742.84)	45.28 (26.1–70.68)	558.84 (322.8–868.54)	27.66 (16.11–42.66)	−1.61 (−1.64 to −1.58)
Low SDI	211.5 (120.62–329.23)	50.57 (28.97–78.45)	340.01 (193.82–529.37)	33.16 (19.01–51.05)	−1.35 (−1.42 to −1.28)
Region					
Central Asia	22.15 (12.49–34.43)	32.77 (18.68–50.54)	25.79 (15.34–39.94)	25.57 (15.26–39.51)	−0.48 (−0.65 to −0.31)
Central Europe	30.59 (17.71–47.13)	24.24 (13.92–37.16)	24.03 (14.26–38.24)	21.81 (12.77–33.78)	−0.26 (−0.4 to −0.13)
Eastern Europe	95.84 (55.53–150.78)	40.97 (23.86–64.05)	81.17 (46.98–127.02)	39.81 (23.2–61.69)	0.31 (0.15 to 0.48)
Australasia	6.16 (3.52–9.65)	28.19 (16.12–44.11)	7.41 (4.38–11.74)	24.73 (14.55–38.79)	−0.27 (−0.34 to −0.19)
High-income Asia Pacific	64.86 (37.24–100.67)	34.19 (19.25–52.9)	50.45 (30.75–76.55)	28.85 (17.87–42.82)	−0.66 (−0.77 to −0.55)
High-income North America	73.32 (40.97–121.77)	23.43 (13.21–39.02)	54.18 (33.46–83.22)	15.13 (9.39–23.22)	−1.93 (−2.09 to −1.76)
Western Europe	88.56 (50.76–137.48)	22.15 (12.65–34.17)	82.71 (49.01–128.19)	21.03 (12.39–32.16)	−0.08 (−0.1 to −0.05)
Andean Latin America	10.66 (5.9–16.44)	29.94 (16.95–45.63)	14.3 (8.19–22.14)	20.13 (11.53–31.33)	−1.18 (−1.25 to −1.11)
Caribbean	10.12 (5.84–15.74)	28.35 (16.39–43.74)	10.28 (5.91–15.76)	20.91 (11.99–32.03)	−0.92 (−0.95 to −0.89)
Central Latin America	46.19 (26.18–71.85)	29.63 (17–44.47)	53.04 (30.56–81.76)	19.62 (11.29–30.26)	−1.28 (−1.37 to −1.2)
Southern Latin America	11.72 (6.75–17.96)	24.16 (13.93–37.2)	14.57 (8.8–22.32)	20.15 (12.19–30.86)	−0.42 (−0.54 to −0.3)
Tropical Latin America	43.39 (24.57–69.49)	28.37 (16.16–45.15)	57.31 (33.37–86.67)	23.11 (13.38–35.15)	−1.05 (−1.28 to −0.82)
North Africa and the Middle East	137.77 (80.41–214.1)	44.69 (26.04–68.34)	200.76 (116.41–306.98)	29.76 (17.29–45.43)	−1.38 (−1.42 to −1.34)
Southeast Asia	179.57 (102.24–275.65)	39.41 (22.81–61.21)	225.18 (132.94–352.18)	29.51 (17.35–45.95)	−0.87 (−0.9 to −0.83)
South Asia	449.36 (257.04–694.75)	43.98 (25.28–68.71)	537.16 (312.88–830.4)	26.65 (15.59–40.94)	−1.64 (−1.66 to −1.62)
East Asia	372.84 (207.56–605.37)	28.2 (15.95–46.43)	290.05 (170.13–459.94)	18.44 (10.97–29.02)	−1.46 (−1.62 to −1.3)
Oceania	3.24 (1.84–5.11)	53.62 (31.02–85.04)	6.26 (3.54–9.92)	45.24 (25.7–71.95)	−0.54 (−0.56 to −0.52)
Central Sub-Saharan Africa	22.35 (12.92–35.35)	48.52 (28.21–76.79)	37.03 (21.14–57.99)	29.95 (17.11–46.03)	−1.45 (−1.57 to −1.33)
Eastern Sub-Saharan Africa	67.4 (38.3–105.04)	43.13 (24.68–66.99)	105.56 (60.03–162.93)	26.9 (15.24–41.6)	−1.5 (−1.55 to −1.45)
Southern Sub-Saharan Africa	17.65 (10.26–27.07)	35.72 (20.82–54.85)	23.49 (13.39–36.6)	26.99 (15.51–41.81)	−0.88 (−0.9 to −0.86)
Western Sub-Saharan Africa	75.6 (43.16–117.61)	45.95 (26.42–71.19)	148.73 (84.53–229.33)	34.33 (19.69–53.05)	−0.87 (−0.92 to −0.81)

DALYs, disability-adjusted life years; ASDR, age-standardized DALY rate; CI, confidence interval; EAPC, estimated annual percentage change; UI, uncertainty interval.

The global ASIR of endometriosis decreased from 1990 to 2021 (EAPC = −1.01, 95% UI = −1.06 to −0.96), being 59.07 (95% UI = 41.2 to 79.24) in 1990 and 43.64 (95% UI = 30.84 to 58.91) in 2021 per 100,000 individuals (Table 1). The global ASDR of endometriosis was similar to ASIR from 1990 to 2021 (EAPC = −0.99, 95% UI = −1.04 to −0.95), being 34.35 (95% UI = 19.58 to 53.96) in 1990 and 25.37 (95% UI = 14.78 to 38.81) in 2021 per 100,000 individuals (Table 2).

### The burden of endometriosis at the national level

**Figure 2:**
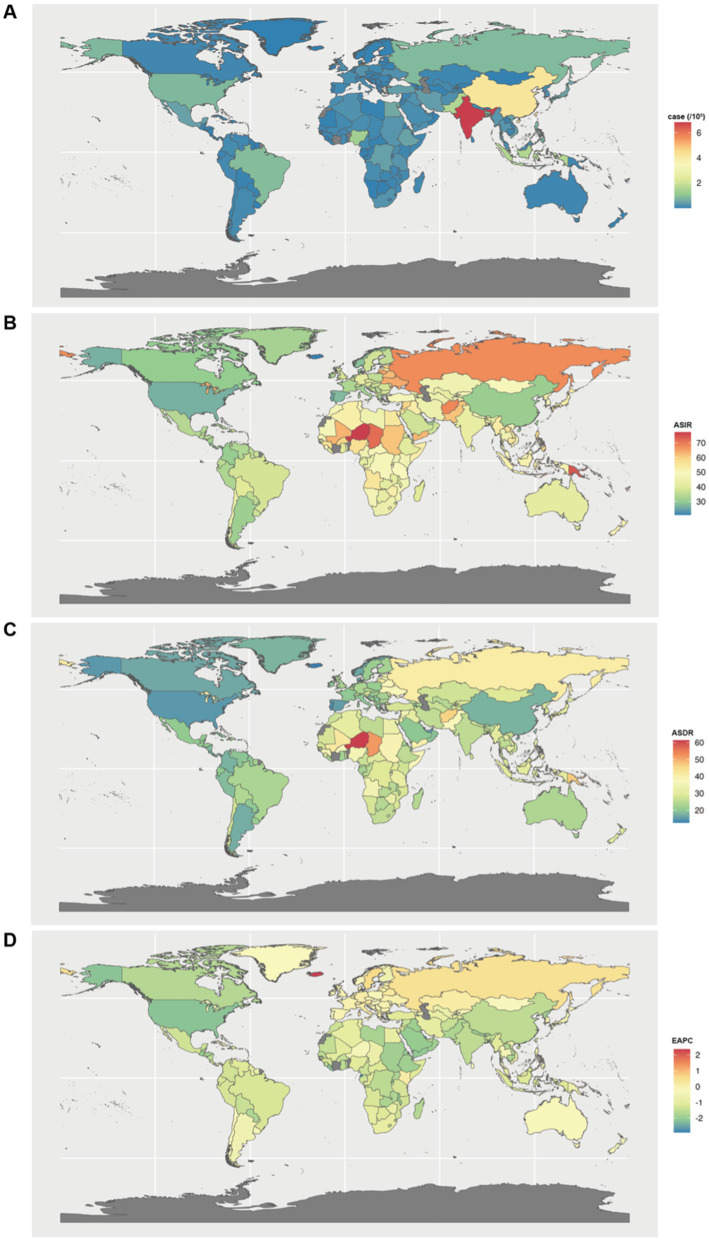
Global disease burden of endometriosis in 204 countries and territories. **(A)** Incident cases in 2021; **(B)** ASIR in 2021; **(C)** ASDR in 2021; **(D)** EAPC from 1990 to 2021. ASIR, age-standardized incidence rate; ASDR, age-standardized rate of disability-adjusted life years; EAPCs, estimated annual percentage changes.

The incidence and DALYs of endometriosis varied considerably at the national level, with the highest cases observed in India (0.68 million and 0.39 million in 2021), followed by China and Indonesia (Figure 2A; Supplementary Figure 1). Incident cases, DALYs, ASIR, and ASDR of endometriosis among 204 countries and territories in 1990 and 2021 are presented in Supplementary Tables 1, 2. Compared to the incident cases in 1990, the largest increase by 2021 was observed in Qatar (324.00% for incidence, 373.33% for DALYs). The decrease by 2021 was most pronounced in the Virgin Islands and Georgia (−60.00% and −50.91% for incidence) and Nauru and the Virgin Islands (−100.00% and −66.67% for DALYs).

The highest ASIR was observed in Niger (77.33 per 100,000 individuals in 2021, 95% UI = 52.74 to 106.78), followed by Solomon Islands and Tokelau (Figure 2B; Supplementary Table 1). From 1990 to 2021, most of the 204 countries and territories experienced a decrease in ASIR. Equatorial Guinea experienced the most significant reduction in ASIR (EAPC = −2.90, 95% UI = −3.03 to −2.78), followed by the Maldives and Qatar. Only four countries and territories—Iceland, Sweden, Austria, and Israel—showed an increase, as shown in Supplementary Figure 2.

The highest ASDR in 2021 was also observed in Niger (61.45 per 100,000 individuals, 95% UI = 34.29 to 95.47), followed by Chad and Solomon Islands (Figure 2C; Supplementary Table 2). The largest decrease in ASDR from 1990 to 2021 was observed in the Maldives (EAPC = −3.17, 95% UI = −3.05 to −3.29), followed by Equatorial Guinea and Yemen. Only six countries and territories that reported an increase in ASDR between 1990 and 2021, as presented in Supplementary Figure 3, were Niger, Iceland, Sweden, Austria, Israel, and Russia.

### The burden of endometriosis at the regional level

**Figure 3:**
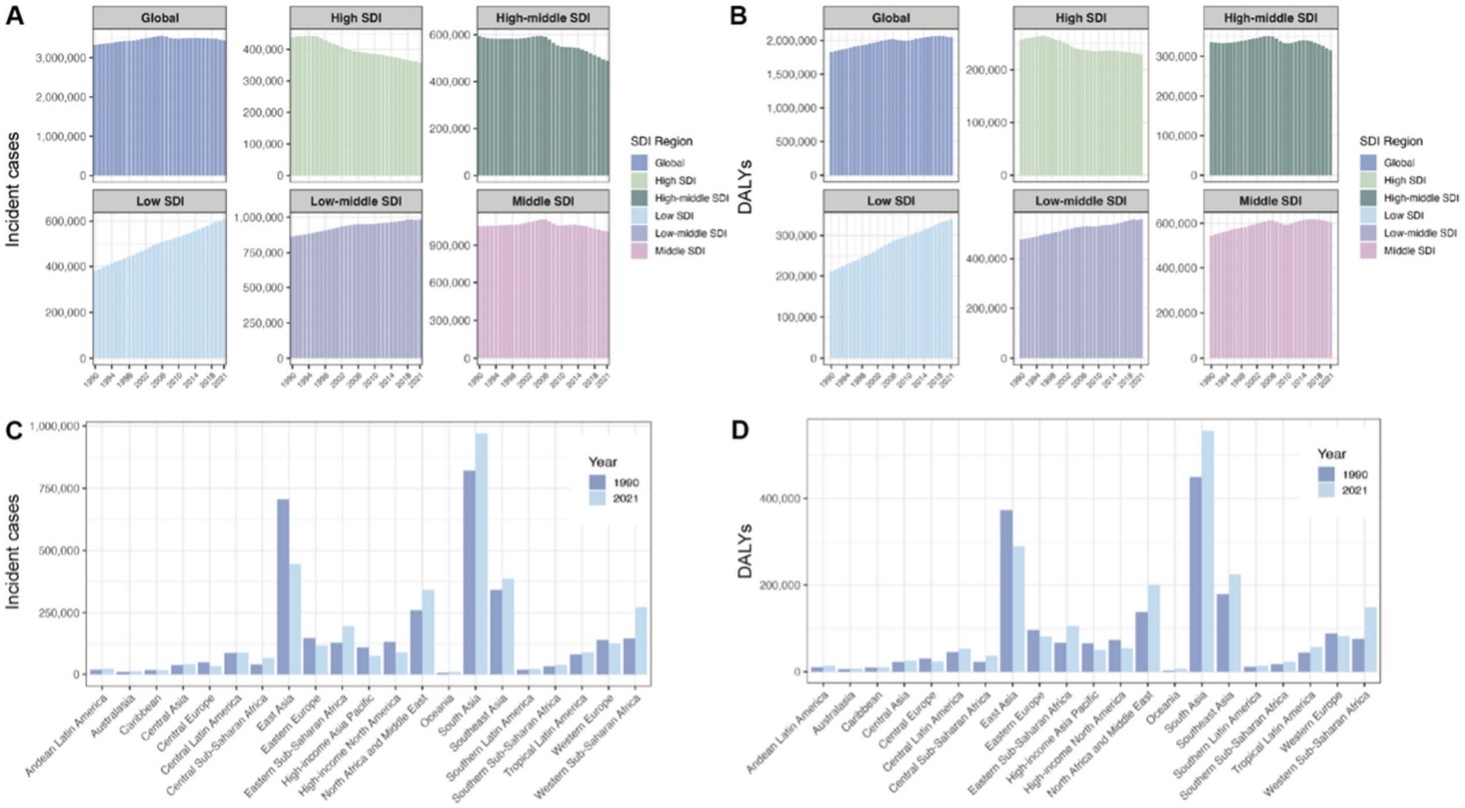
Incident cases and DALYs of endometriosis at SDI and regional levels. **(A)** Incident cases in SDI regions from 1990 to 2021. **(B)** DALYs in SDI regions from 1990 to 2021. **(C)** Incident cases at the regional level in 1990 and 2021. **(D)** DALYs at the regional level in 1990 and 2021.

The number of incident cases and DALYs differed among the 21 regions (Figures 3C,D). Overall, the incident cases and DALYs in 2021 were the highest both in South Asia (936,790, 95% UI = 643,280 to 1,268,520; and 537,160, 95% UI = 312,880 to 830,400; respectively). The lowest cases and DALYs were in Oceania (10,520, 95% UI = 7,360 to 14,570; and 6,260, 95% UI = 3,540 to 9,920; respectively). At the regional level, the changes in the two indices between 1990 and 2021 were similar. Between 1990 and 2021, the incident cases increased in 14 regions and decreased in 7 regions. For DALYs, 15 regions showed an increase, while 6 regions showed a decrease. Compared to 1990, East Asia showed the largest decrease in 2021 (−36.88% for incidence, −22.21% for DALYs), while South Asia showed the largest increase in 2021 (16.95% for incidence, 19.54% for DALYs), respectively (Tables 1, 2). The six regions with a reduction in DALYs are Central Europe, Eastern Europe, High-income Asia Pacific, High-income North America, Western Europe, and East Asia. Notably, the Caribbean was the only region where the incident cases decreased (18,430 in 1990, 17,090 in 2021) while DALYs slightly increased (10,120 in 1990, 10,280 in 2021).

**Figure 4:**
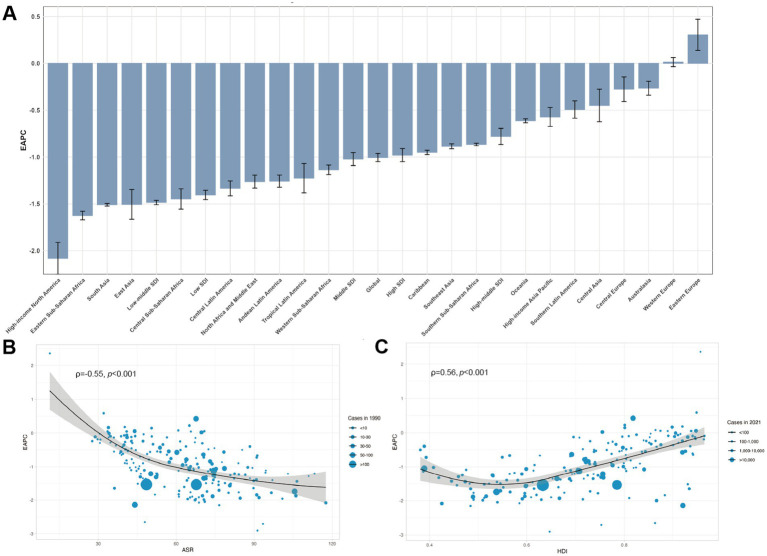
EAPCs of endometriosis at the global, regional, and national levels. **(A)** The EAPC of endometriosis from 1990 to 2021. **(B)** The correlation between EAPC and endometriosis ASR in 2021. **(C)** The correlation between EAPC and HDI in 2021. The circles represent countries that were available on HDI data. The size of circle is increased with the cases of endometriosis. The *ρ* indices and *p*-values presented in B and C were derived from Pearson’s correlation analysis. ASR, age-standardized rate; EAPC, estimated annual percentage change; HDI, human development index.

The highest ASIR and ASDR in 21 regions were both in Oceania (77.71 per 100,000 individuals for ASIR, 95% UI = 51.23 to 100.27; 45.24 per 100,000 individuals for ASDR, 95% UI = 45.24 to 71.95), while the lowest were both in High-income North America (26.90 per 100,000 individuals for ASIR, 95% UI = 19.19 to 35.32; 15.13 per 100,000 individuals for ASDR, 95% UI = 9.39 to 23.22), respectively. From 1990 to 2021, the ASIR and ASDR indices decreased across all regions, with the largest decline observed in high-income North America (EAPC = −2.08 for ASIR, 95% UI = −2.25 to −1.91; EAPC = −1.9 for ASDR, 95% UI = −2.09 to −1.76), respectively (Tables 1, 2; Figure 4A).

### The burden of endometriosis at the SDI region level

At the SDI regional level, the incident cases in the high SDI region demonstrated a marked decline from 1990 to 2021, while cases in the low SDI region exhibited a significant upward trend. The high-middle SDI region initially experienced a plateau from 1990 to 2006, followed by a gradual decrease until 2021. In contrast, the incident cases in the middle SDI region remained relatively stable over the same period, whereas the low-middle SDI region showed a modest upward trend (Figure 3A). DALYs in the high SDI region followed a decreasing trend, in contrast to a pronounced increase observed in the low SDI region. The high-middle SDI region remained relatively stable throughout the period, while both the low-middle and middle SDI regions showed a slight upward trend (Figure 3B).

The highest ASR in 2021 among all the SDI regions was low SDI (52.42 per 100,000 individuals for incidence, 95% UI = 26.19 to 47.95; 33.16 per 100,000 for DALYs, 95% UI = 19.01 to 51.05), while the lowest was high SDI (36.44 per 100,000 individuals for incidence, 95% UI = 47.95 to 26.19; 21.24 per 100,000 individuals for DALYs, 95% UI = 12.80 to 32.29), respectively. The largest decrease in ASIR and ASDR among all SDI regions from 1990 to 2021 was both low-middle SDI (EAPC = −1.49, 95% UI = −1.51 to −1.46; EAPC = −1.61, 95% UI = −1.64 to −1.58), respectively (Tables 1, 2; Figure 4A).

### Relationship between EAPC and economic health indicators

We analyzed the relationships between EAPC and ASIR in 1990, as well as HDI in 2021, across 204 countries and territories (Figures 4B,C). The ASIR of endometriosis in 1990 serves as an indicator of the baseline disease burden, while the HDI in 2021 provides a proxy for each country’s healthcare level and accessibility. A significant negative correlation was identified between EAPC and ASIR (*ρ* = −0.55, *p* < 0.001), indicating that regions with higher ASIRs tended to have slower growth rates over time. In addition, a significant positive association was observed between EAPC and HDI (*ρ* = 0.56, *p* < 0.001), suggesting that countries and territories with higher HDI experienced a more rapid increase in ASIR of endometriosis from 1990 to 2021.

## Discussion

Endometriosis has a large global burden, which can negatively affect all aspects of a patient’s daily life (10). The prevalence of endometriosis is highest in women with infertility or chronic pelvic pain, reaching 25–50% and 71–87%, respectively (11, 12). Previous studies have suggested a certain degree of decline in the prevalence and disease burden of endometriosis (13); however, recent literature indicates that this trend may be reversing (6). It is crucial to update and analyze the current situation to provide a basis for public health policy formulation. Here, our study detailed the incidence, DALYs, and their age-standardized rates of endometriosis across 204 countries and territories from 1990 to 2021 at the global, regional, and national levels. Based on the GBD 2021 study, this comprehensive analysis offers crucial insights into trends in the burden of endometriosis across various demographics and regions, highlighting an urgent need for more effective and targeted interventions.

Our study reveals that 3.45 million persons were suffering from endometriosis in 2021, which represented an increase of 3.51%. The DALYs associated with endometriosis showed a notable increase from 1990 to 2021, rising from 1.83 million to 2.05 million, representing an approximate 12.03% growth. We estimate that the actual burden of endometriosis may be underestimated due to delays in diagnosis. A definitive diagnosis, typically confirmed by laparoscopic surgery, takes an average of 7–9 years (14, 15). The increasing global incidence of endometriosis is largely attributed to improved diagnostic capabilities and heightened awareness, particularly in well-developed regions. In addition to improved diagnostic capabilities and population growth, environmental factors, lifestyle changes (such as delayed childbearing and hormone use), and socioeconomic factors may also contribute to changes in disease burden. Further investigation into these potential factors would provide a more comprehensive understanding of the epidemiological characteristics of endometriosis.

Globally, endometriosis is particularly prevalent in South Asia and East Asia, with a high incidence in countries such as India and China. In 2021, India and China together accounted for 32.35% of the total DALYs attributed to endometriosis and 20.08% of global incident cases. In Qatar and the United Arab Emirates, the incidence has increased by 4.20- and 2.86-fold, respectively, compared to 1990 levels. This rise may be attributed to advancements in healthcare infrastructure and access, as well as rapid population growth and demographic shifts. The pronounced decrease in endometriosis cases observed in the Virgin Islands and Georgia may be largely attributed to a declining population in these regions. Compared to 1990, DALYs in the majority of the 204 countries and territories increased by 2021, indicating that the disease burden of endometriosis has not been alleviated, while a few regions, such as Central Europe, Eastern Europe, High-income Asia Pacific, High-income North America, Western Europe, and East Asia, experienced a reduction, which may be related to economic development.

While the incidence cases of endometriosis and the resulting DALYs are high, the ASIR and ASDR globally had even slightly decreased. In addition, the ASIR and ASDR varied significantly across regions, countries, and territories, with Oceania showing the highest rates, mainly due to contributions from the Solomon Islands and Tokelau. However, in contrast to the high incidence, the ASIR and ASDR in India and China were not very high, which might be mainly attributed to the large population base. As for Iceland and Sweden, the observed increases in ASIR may partly reflect improved case ascertainment due to enhanced awareness, better access to specialized diagnostic tools, and more consistent use of laparoscopic evaluation, rather than a true increase in disease incidence. The largest reduction in ASIR and ASDR from 1990 to 2021 was observed in high-income North America, likely due to improvements in treatment options for endometriosis, such as medication, minimally invasive surgery, and hormone therapy, along with early diagnosis that helps prevent disease progression and reduce both incidence and disability rates. Although the decreasing trends in ASIR and ASDR indicate a reduction in the incidence and mortality rates per individual, the overall global disease burden (i.e., the incidence and overall health loss) continues to rise.

The higher incidence and DALYs among younger individuals, especially those aged 20–24 and 25–29, reflect the estrogen-dependent nature of endometriosis (14). Similarly, Chapron et al. observed a higher frequency of the DIE phenotype and a lower prevalence of isolated superficial lesions in patients older than 24 years compared to those aged 24 years or younger. They also found that, between the ages of 25 and 42, the distribution of endometriosis phenotypes remained stable (16). In addition, aging populations in regions such as Eastern Europe and High-income Asia Pacific contribute to a natural reduction in endometriosis incidence as the condition primarily affects women of reproductive age. To effectively address the age-specific incidence of endometriosis, public health policies should emphasize early screening programs targeting women of reproductive age and provide comprehensive support services, including psychological and pain management (17). Specific regional interventions are also needed, particularly in areas experiencing significant population aging, to adapt healthcare resources to the changing needs of women of reproductive age. In addition, hormone management strategies should be implemented to modify the hormonal environment, either by suppressing ovarian activity (including the secretion of endocrine sex steroids) or by targeting steroid receptors and enzymes within the endometrium and lesions. These approaches aim to reduce the risk of disease progression effectively (14).

Another important finding of this study is the significant association between SDI and the disease burden of endometriosis. The ASIR and ASDR were higher in the low and low-middle SDI regions than the global average. In addition, the incident cases and DALYs in the low SDI region showed a pronounced upward trend, contrasting with a downward trend observed in the high SDI region. This finding may suggest that poverty is a potential risk factor for endometriosis. Low SDI regions typically have rapid population growth and a relatively high proportion of young women. This demographic structure naturally leads to higher incidence rates and overall burden. More importantly, the aggravated burden in low-SDI areas underscores the critical deficiencies in healthcare resources, health education, and public health interventions. Future efforts should prioritize enhancing healthcare accessibility, early screening, and effective treatment in low-SDI regions, while high-SDI regions should focus on optimizing disease management to reduce morbidity and adverse outcomes. These disparities underscore the need for region-specific strategies to alleviate the global burden of endometriosis and improve public health outcomes. In low-SDI regions, recommended strategies include strengthening the training of primary healthcare providers—particularly in minimally invasive diagnostic techniques—and implementing task-sharing approaches to expand surgical capacity. In addition, increasing public awareness of endometriosis and improving access to diagnostic tools are essential. In high-SDI regions, efforts should focus on refining existing treatment protocols to reduce disease recurrence and enhance patients’ quality of life.

Our study found a significant negative association between EAPC and baseline ASR (<10/100,000) from 1990 to 2021. Countries or regions with lower baseline ASR showed a larger relative increase in endometriosis incidence, likely because it was not initially prioritized as a public health issue due to its low burden. In contrast, the degree of difficulty in controlling endometriosis increased with a higher baseline disease burden, making it harder to reduce incidence in regions with already high ASR. The positive correlation between EAPC and HDI suggests that countries with higher healthcare accessibility and quality experienced faster growth in the incidence rate of endometriosis. This may be attributed to improved screening and diagnostic capabilities in these high-HDI regions, leading to increased identification of cases.

Importantly, while age-standardized incidence and death rates (ASIR and ASDR) have decreased in some high-HDI countries, the absolute number of cases has paradoxically increased. This reflects enhanced diagnostic access and greater awareness, resulting in more cases being detected rather than an actual rise in disease occurrence. Such a phenomenon underscores the need for healthcare systems to consider both relative rates and absolute case counts when planning resources, as rising absolute numbers translate into increased demands for diagnosis, treatment, and patient management despite declining standardized rates.

Endometriosis is associated with infertility. It is observed in 50–80% of women with pelvic pain and up to 50% of women with infertility (5). Notably, approximately 10% of ART patients have endometriosis. Given the potential for ovarian damage, women with endometriosis—particularly those with bilateral endometriomas or prior recurrent ovarian surgeries—should receive counseling regarding the risk of diminished ovarian reserve (18). Preoperative fertility preservation strategies can mitigate the adverse impact of surgical interventions on ovarian reserve. Recent studies have demonstrated that several micronutrients and vitamins, including betaine, myo-inositol, and vitamin D, may play a significant role in women’s health, particularly in breast cancer prevention and polycystic ovary syndrome (PCOS). Moreover, the relationship between lifestyle, nutrition, and gynecological conditions such as endometriosis and early-stage endometrial cancer has gained attention, indicating the potential for targeted prevention strategies based on nutritional and molecular profiling (19–26).

Although the GBD estimates fill a gap where actual data on disease burden are sparse or unavailable, several limitations should be noted. We acknowledge that the analytical methods applied may be limited in uncovering deeper associations or localized patterns due to the constraints of aggregate-level modeling. Future research could incorporate more innovative approaches, such as machine learning or spatiotemporal modeling, to uncover more nuanced patterns and relationships in endometriosis epidemiology. In addition, the accuracy of these data is highly dependent on the quality of medical record-keeping and reporting systems in individual countries. In low-SDI regions, where healthcare infrastructure is limited, data collection may be substantially biased, potentially resulting in inaccurate estimates of the incidence and disease burden of endometriosis. Second, the diagnosis of endometriosis typically requires laparoscopic surgery—a procedure with widely varying accessibility across regions—which may lead to significant underestimation of the true incidence. Third, although the study assesses the disease burden of endometriosis at the global, regional, and national levels, it does not provide an in-depth analysis of specific high-risk populations, such as women experiencing infertility or chronic pelvic pain. These groups are known to have a higher incidence of endometriosis and may face a distinct disease burden compared to the general population. Future research should further investigate incidence patterns and disease burden within these specific populations to better guide clinical practice and inform targeted public health policies. In addition, a considerable portion of the societal burden arises from endometrioma and deep infiltrating endometriosis. However, due to limited data, the study does not analyze the incidence trends of these subtypes, potentially leading to an incomplete understanding of the overall disease burden. Future research could incorporate subtype data to provide a more accurate assessment of the disease burden. Additionally, advanced analytical tools—such as machine learning algorithms, spatial–temporal modeling, or network analysis— could be employed to uncover more complex patterns and predictors of disease burden.

## Conclusion

This study utilized the GBD 2021 database to analyze the disease burden of endometriosis across various countries and regions globally. While the global ASIR has decreased, the absolute number of cases and DALYs in 2021 remains high, highlighting the need for continued attention to the global burden of endometriosis. The findings highlight disparities across different SDI regions. Effective management of endometriosis necessitates region-specific interventions, early screening, improved healthcare accessibility, and public awareness campaigns to address the persistent global disease burden. Managing this condition remains a significant challenge, and future research should focus on identifying underlying risk factors and developing innovative treatment options to address this widespread and impactful condition.

## Data Availability

The original contributions presented in the study are included in the article/Supplementary material, further inquiries can be directed to the corresponding author.

## Footnotes

^1^ https://ghdx.healthdata.org/record/ihme-data/gbd-2021-cause-icd-code-mappings

^2^ https://ghdx.healthdata.org/gbd-2021/code/nonfatal-5

^3^ https://vizhub.healthdata.org/gbd-results/

^4^ https://hdr.undp.org/data-center/human-development-index#/indicies/HDI

^5^ https://www.r-project.org/
