# Supplementary material for: Global, regional, and national burdens of endometriosis from 1990 to 2021: a trend analysis
Source: Front Med (Lausanne). 2025 Aug 14;12:1562196. doi: 10.3389/fmed.2025.1562196 (PMC12391101; doi:10.3389/fmed.2025.1562196)

**Supplemental Material**

**Content**

Supplemental Figure 1......................................................................................................2

Supplemental Figure 2......................................................................................................2

Supplemental Figure 3......................................................................................................2

Supplemental Figure 4......................................................................................................3

Supplemental Figure 5......................................................................................................3

Supplemental Figure 6......................................................................................................3

Supplemental Table 1........................................................................................................4

Supplemental Table 2......................................................................................................20

Supplemental Table 3......................................................................................................36

Supplemental Appendix 1...............................................................................................36

Supplemental Appendix 2...............................................................................................37


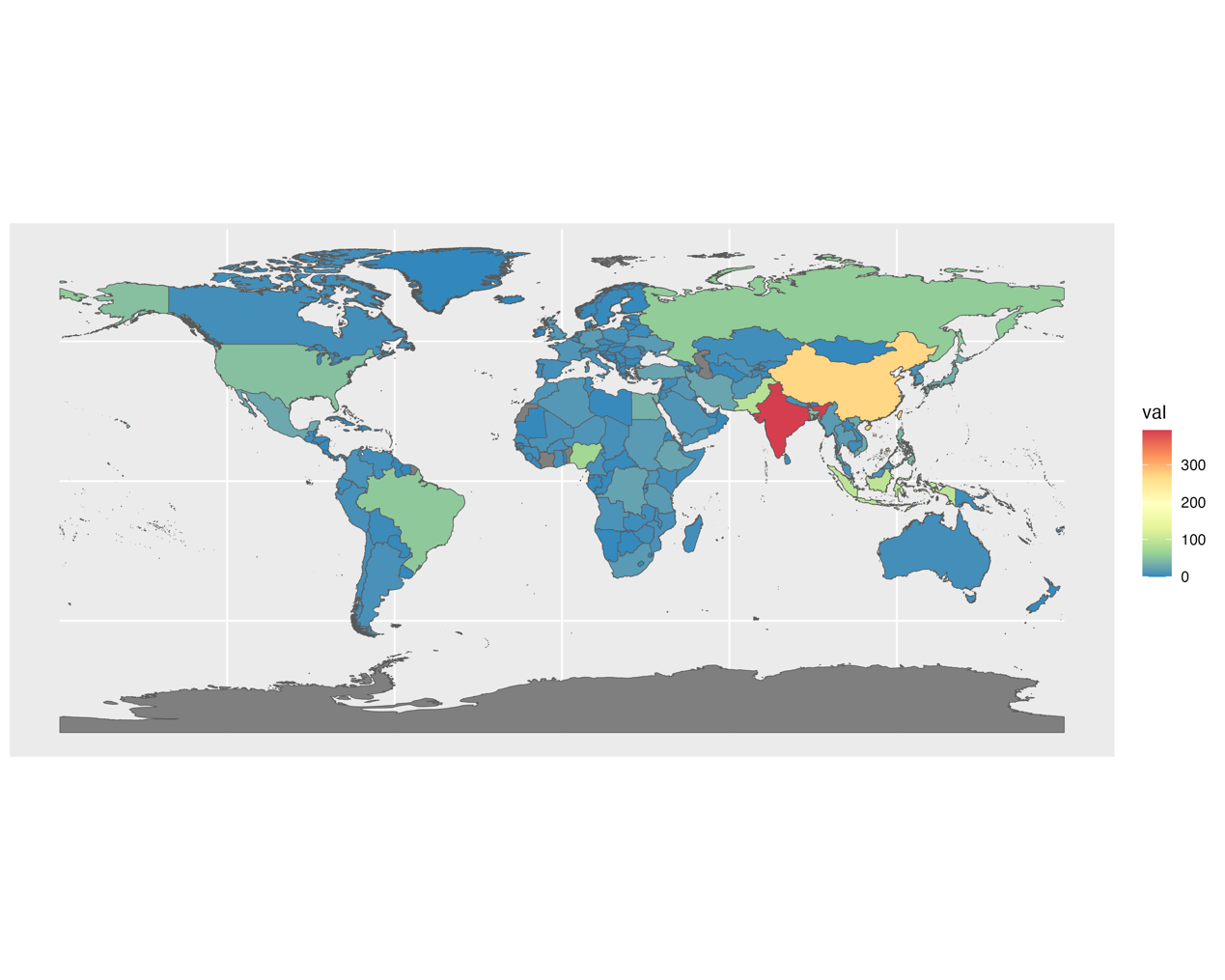


**Supplemental Figure 1.** The global DALYs in 204 countries and territories in 2021.


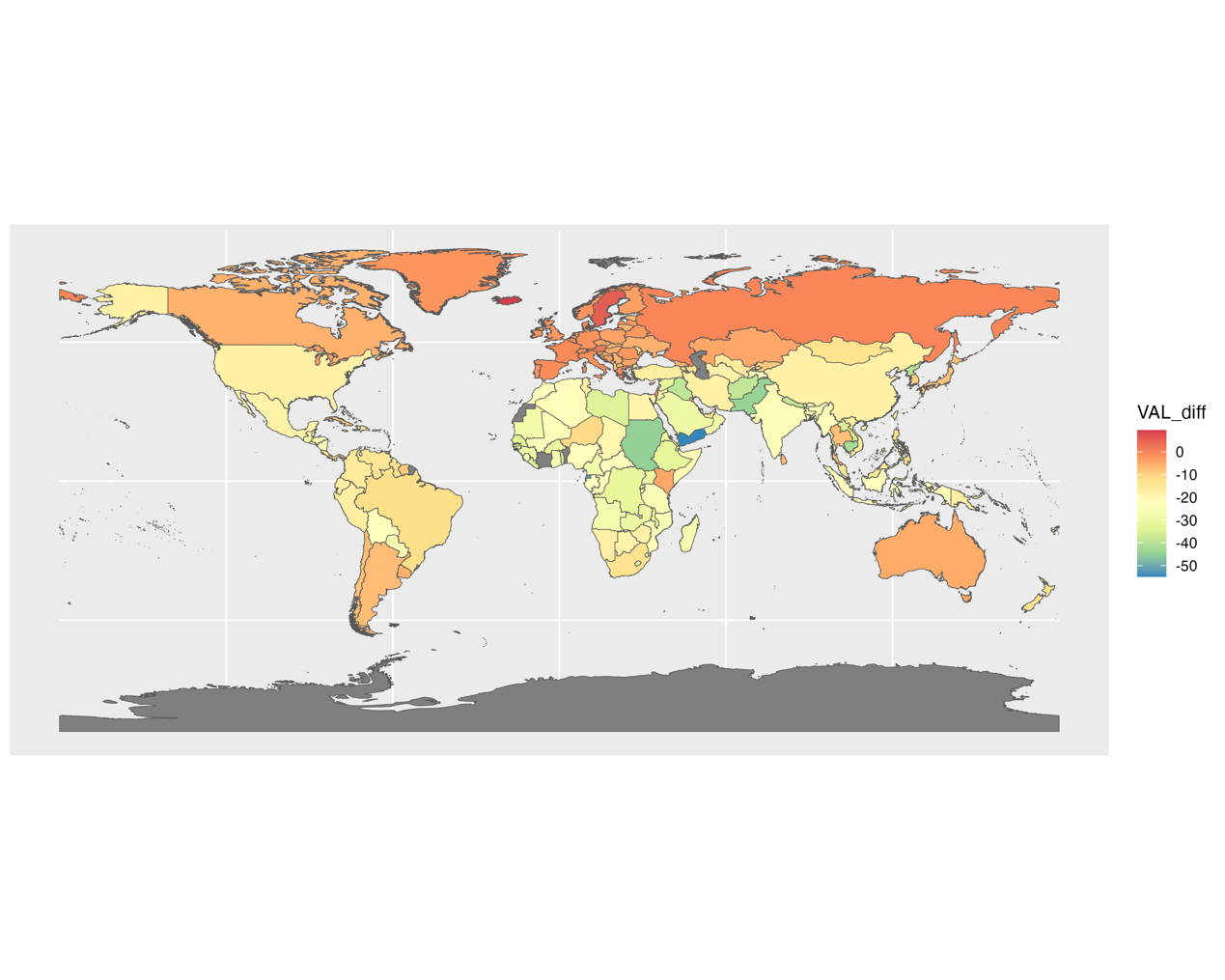


**Supplemental Figure 2.** The Change of ASIR between 1990 and 2021 in 204 countries and territories.


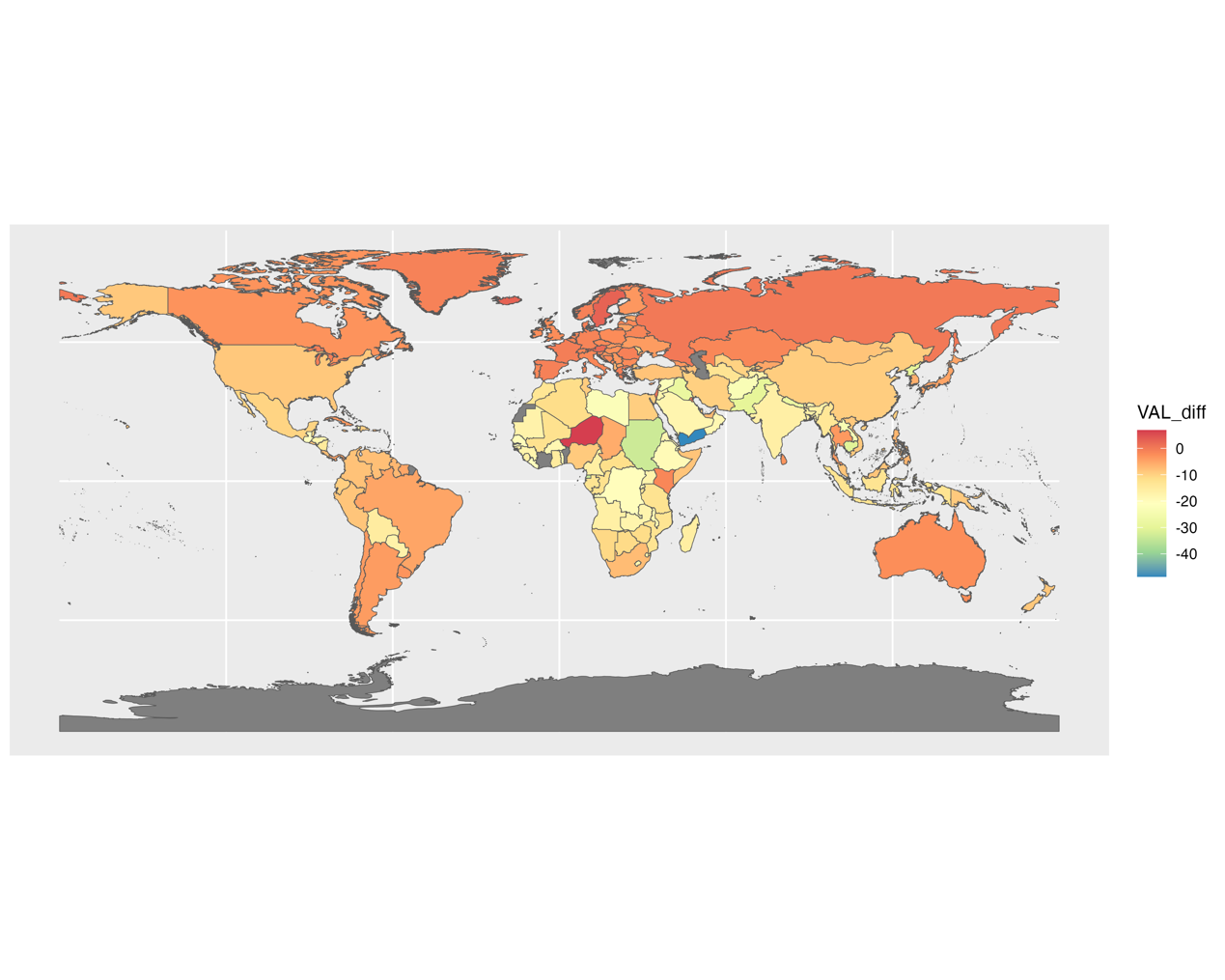


**Supplemental Figure 3.** The Change of ASDR between 1990 and 2021 in 204 countries and territories.


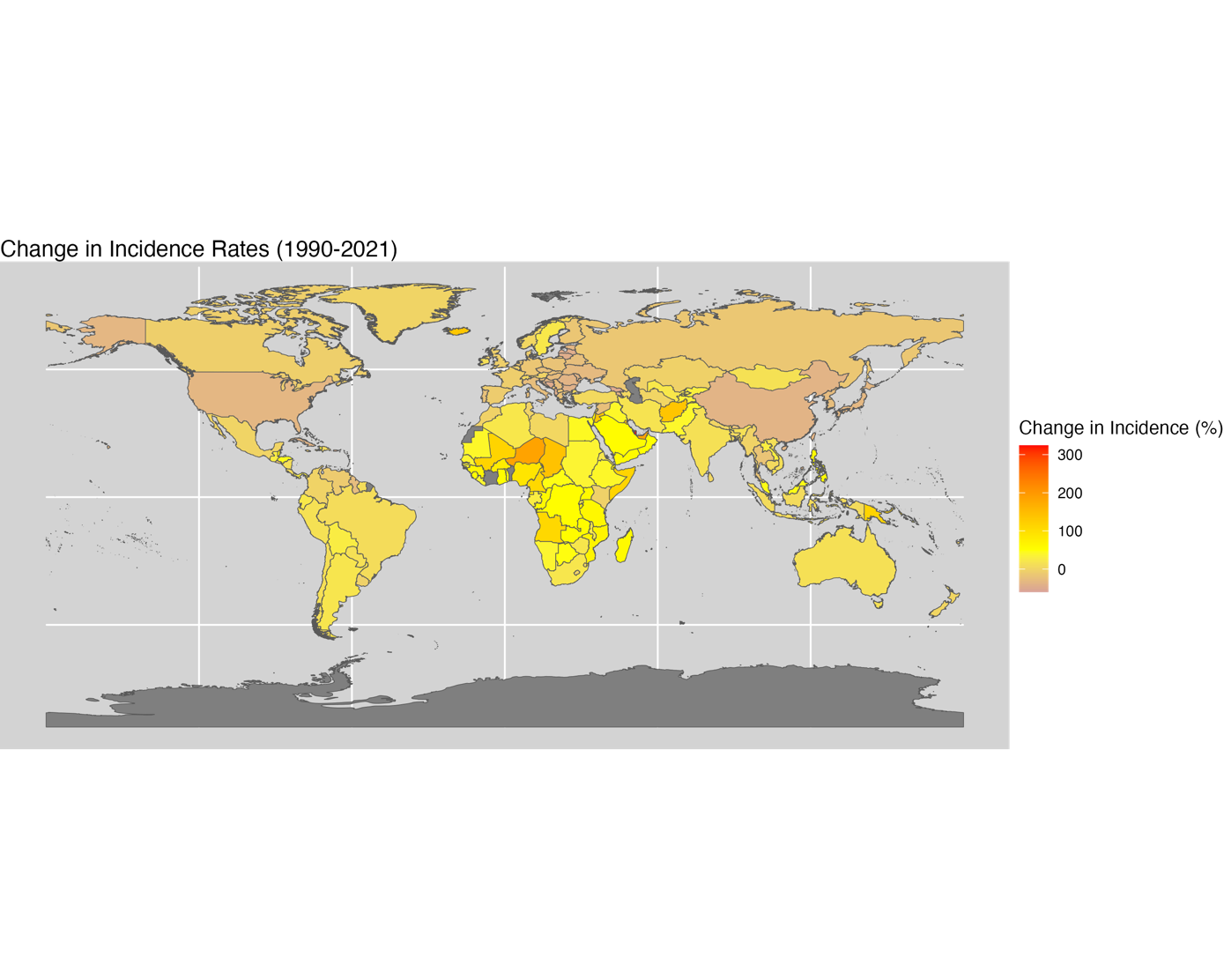


**Supplemental Figure 4.** The relative change(%) in incident cases of endometriosis between 1990 and 2021 in 204 countries and territories.


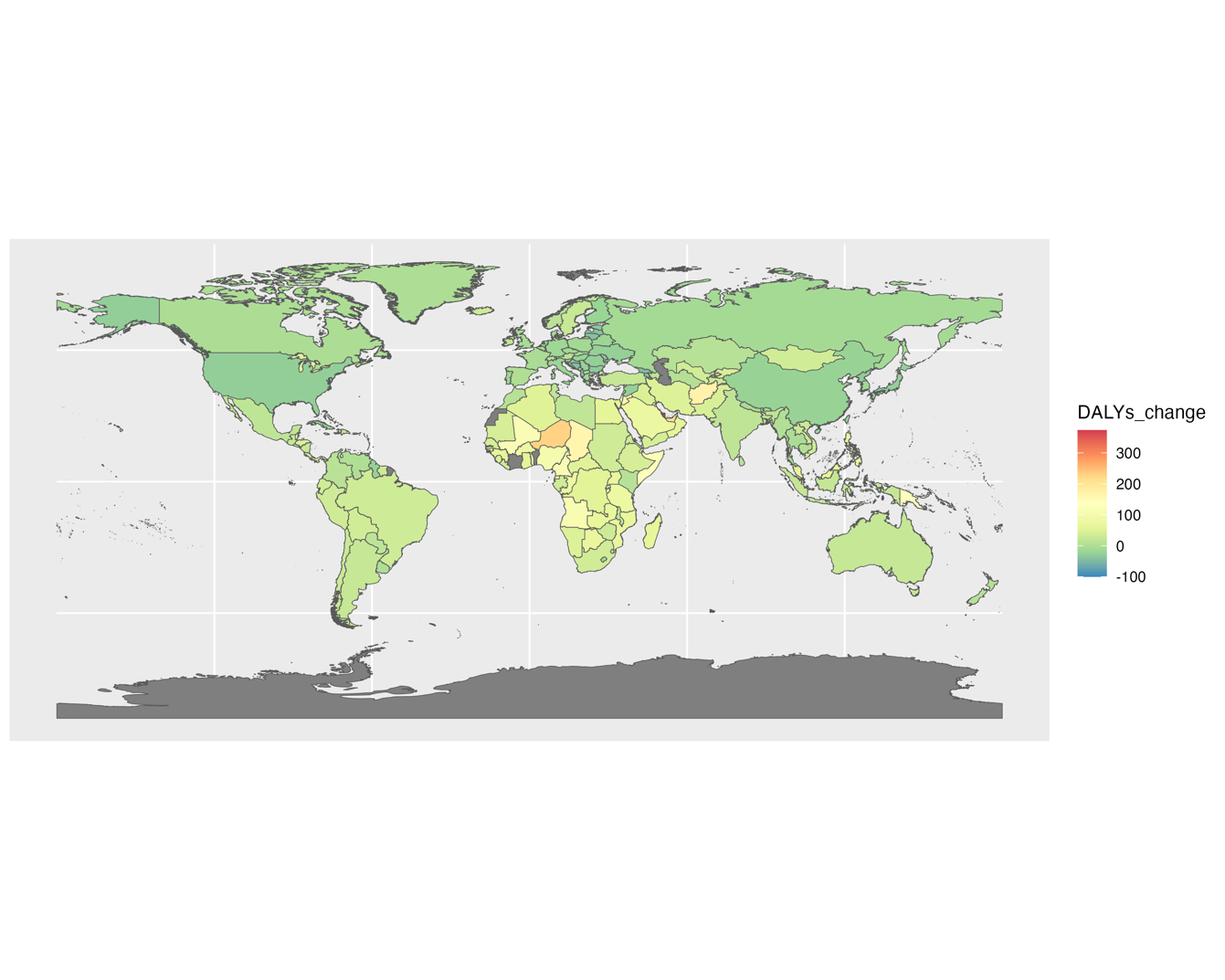


**Supplemental Figure 5.** The relative change(%) in DALYs of endometriosis between 1990 and 2021 in 204 countries and territories.


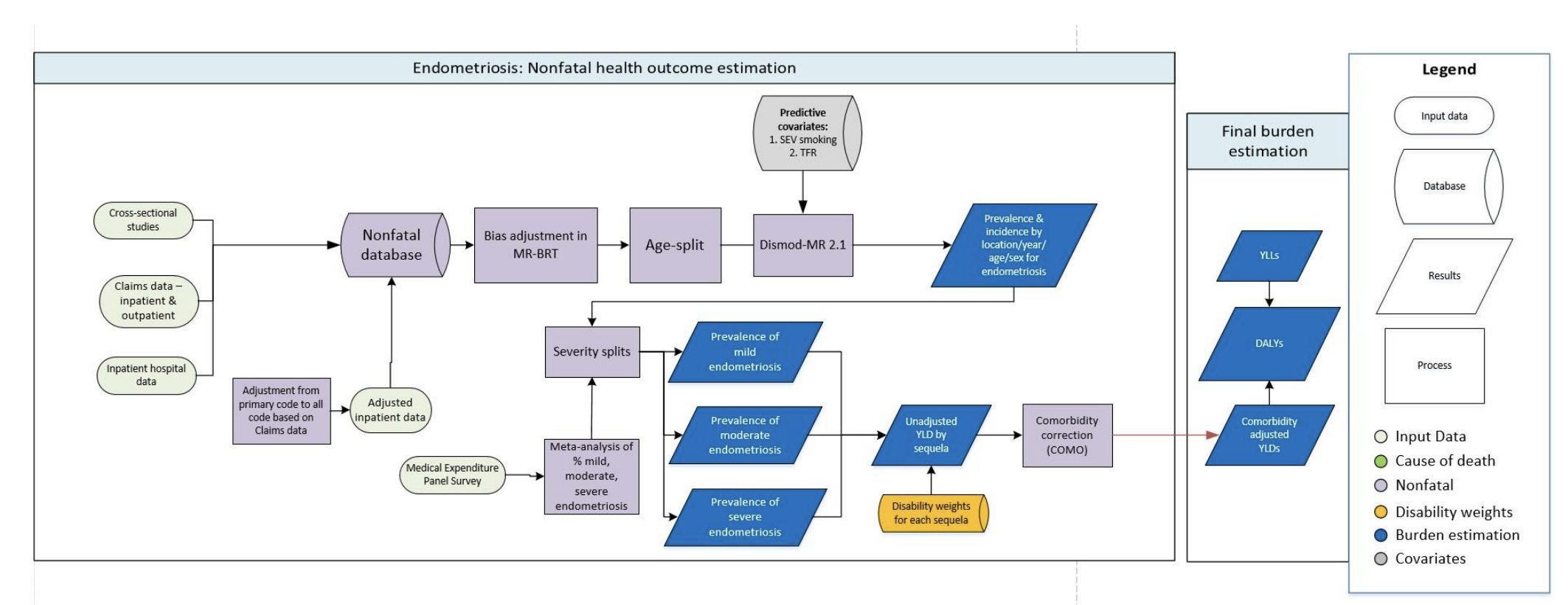


**Supplemental Figure 6.** The flowchart of endometriosis in GBD 2021.

**Supplemental Table 1.** The incideny cases and ASIR of endometriosis in 1990 and 2021, and its temporal trends from 1990 to 2021 in 204 countries and territories.

| Characteristics | 1990 | | 2021 | | 1990-2021 |
| --- | --- | --- | --- | --- | --- |
|  | Cases No.$\times$10^3^ (95%UI) | ASIR per 100,000 No. (95%UI) | Case No.$\times$10^3^ (95%UI) | ASIR per 100,000 No. (95%UI) | EAPC  No. (95% UI) |
| American Samoa | 0.05 (0.03 - 0.06) | 89.34 (61.69 - 118.97) | 0.03 (0.02 - 0.04) | 63.9 (44.03 - 87.77) | -1.03  (-1.05 - -1.01) |
| Antigua and Barbuda | 0.03 (0.02 - 0.04) | 42.17 (29.37 - 57.49) | 0.03 (0.02 - 0.04) | 31.68 (21.96 - 43.5) | -1.03  (-1.07 - -0.98) |
| Arab Republic of Egypt | 41.53 (28.12 - 58.59) | 73.07 (50.75 - 100.09) | 59.6 (41.22 - 82.39) | 54.25 (38.01 - 74.58) | -0.84  (-0.91 - -0.77) |
| Argentine Republic | 12.47 (8.61 - 16.99) | 38.75 (26.66 - 52.77) | 15 (10.34 - 20.18) | 31.55 (21.73 - 42.38) | -0.55  (-0.62 - -0.49) |
| Australia | 8.43 (5.93 - 11.63) | 47.21 (32.99 - 65.14) | 9.63 (6.62 - 12.94) | 42.08 (28.97 - 57.44) | -0.24  (-0.31 - -0.17) |
| Barbados | 0.11 (0.07 - 0.15) | 39 (27.25 - 52.21) | 0.09 (0.06 - 0.12) | 31.26 (21.07 - 42.54) | -0.65  (-0.71 - -0.59) |
| Belize | 0.1 (0.06 - 0.14) | 52.32 (36.43 - 71.01) | 0.17 (0.12 - 0.24) | 34.95 (23.94 - 48.3) | -1.2  (-1.26 - -1.14) |
| Bermuda | 0.03 (0.02 - 0.03) | 38.34 (26.45 - 51.78) | 0.02 (0.01 - 0.02) | 31.8 (22.01 - 44.68) | -0.6  (-0.67 - -0.53) |
| Bolivarian Republic of Venezuela | 9.6 (6.59 - 13.51) | 47.73 (33.23 - 65.51) | 8.61 (5.98 - 11.68) | 33.72 (23.1 - 45.63) | -1.1  (-1.21 - -1) |
| Bosnia and Herzegovina | 1.97 (1.37 - 2.7) | 40.36 (28.23 - 55.02) | 1.04 (0.74 - 1.41) | 36.91 (25.98 - 50.49) | -0.26  (-0.34 - -0.18) |
| Brunei Darussalam | 0.19 (0.13 - 0.26) | 64.57 (42.94 - 86.22) | 0.25 (0.17 - 0.34) | 47.23 (32.28 - 63.06) | -1.02  (-1.05 - -0.98) |
| Burkina Faso | 7.32 (5.01 - 10.26) | 89.85 (61.25 - 122.35) | 13.07 (8.87 - 18.04) | 59.02 (40.33 - 79.43) | -1.43  (-1.46 - -1.39) |
| Canada | 10.84 (7.37 - 14.92) | 36.23 (24.45 - 50.22) | 10.15 (7.13 - 13.61) | 30.44 (21.2 - 40.7) | -1.65  (-2.32 - -0.97) |
| Central African Republic | 1.92 (1.29 - 2.69) | 72.29 (49.7 - 99.41) | 2.83 (1.93 - 4.05) | 49.93 (34.57 - 69.19) | -1.14  (-1.18 - -1.1) |
| Commonwealth of Dominica | 0.03 (0.02 - 0.05) | 46.4 (32.4 - 63.26) | 0.02 (0.01 - 0.03) | 31.46 (22.04 - 42.98) | -1.21  (-1.28 - -1.14) |
| Commonwealth of the Bahamas | 0.12 (0.08 - 0.17) | 40.29 (27.83 - 55.06) | 0.13 (0.09 - 0.18) | 31.25 (21.16 - 42.59) | -0.89  (-0.92 - -0.86) |
| Cook Islands | 0.01 (0.01 - 0.02) | 74.76 (52.13 - 100.86) | 0.01 (0.01 - 0.01) | 63.51 (44.05 - 86.1) | -0.45  (-0.52 - -0.37) |
| Czech Republic | 4.44 (3.18 - 6.01) | 44.23 (31.39 - 59.95) | 3.35 (2.35 - 4.53) | 39.95 (27.63 - 54.7) | -0.09  (-0.26 - 0.08) |
| Democratic People's Republic of Korea | 19.95 (14.51 - 27.05) | 91.03 (65.8 - 121.72) | 14.24 (10.27 - 19.4) | 51 (36.28 - 69.17) | -1.85  (-1.95 - -1.74) |
| Democratic Republic of Sao Tome and Principe | 0.07 (0.05 - 0.1) | 66.19 (45.71 - 89.04) | 0.09 (0.06 - 0.13) | 38.07 (26.6 - 52.86) | -1.9  (-1.97 - -1.82) |
| Democratic Republic of the Congo | 29.16 (19.73 - 40.79) | 80.7 (56.07 - 109.4) | 44.75 (30.24 - 63.16) | 48.41 (32.66 - 66.44) | -1.57  (-1.69 - -1.45) |
| Democratic Republic of Timor-Leste | 0.76 (0.53 - 1.04) | 94.36 (66.27 - 126.89) | 0.98 (0.69 - 1.36) | 67.28 (47.54 - 91.65) | -1.18  (-1.27 - -1.09) |
| Democratic Socialist Republic of Sri Lanka | 10.38 (7.29 - 14.15) | 54.89 (38.94 - 73.43) | 10.62 (7.53 - 14.11) | 48.02 (34.02 - 63.88) | -0.34  (-0.38 - -0.3) |
| Dominican Republic | 4.09 (2.82 - 5.8) | 52.6 (36.88 - 71.76) | 4.39 (3.01 - 6.08) | 36.85 (25.27 - 51.04) | -1.21  (-1.28 - -1.14) |
| Eastern Republic of Uruguay | 1.28 (0.87 - 1.72) | 42.8 (28.78 - 57.41) | 1.23 (0.84 - 1.66) | 37.17 (25.48 - 50.01) | -0.45  (-0.5 - -0.41) |
| Federal Democratic Republic of Ethiopia | 34.46 (23.73 - 47.61) | 74.05 (51.32 - 99.01) | 48.55 (33.49 - 67.79) | 41.19 (28.83 - 56.29) | -1.89  (-1.93 - -1.84) |
| Federal Democratic Republic of Nepal | 15.99 (10.98 - 22.21) | 84.66 (57.35 - 115.14) | 16.16 (11.13 - 22.42) | 46.38 (32.33 - 63.55) | -1.94  (-2.04 - -1.84) |
| Federal Republic of Germany | 33.93 (23.02 - 47.22) | 44.03 (30.4 - 60.96) | 28.47 (19.52 - 38.76) | 42.04 (29.01 - 58.15) | 0.06  (-0.03 - 0.14) |
| Federal Republic of Nigeria | 70.15 (48.02 - 98.2) | 78.59 (54.27 - 106.57) | 132.56 (91.43 - 185.6) | 56.84 (39.28 - 76.76) | -1.05  (-1.11 - -0.99) |
| Federal Republic of Somalia | 5.33 (3.62 - 7.43) | 73.22 (49.38 - 100.56) | 11.46 (7.75 - 15.86) | 52.73 (35.91 - 71.87) | -1.08  (-1.15 - -1.02) |
| Federated States of Micronesia | 0.1 (0.07 - 0.13) | 97.05 (68.91 - 131.92) | 0.07 (0.05 - 0.09) | 61.42 (42.49 - 84.55) | -1.41  (-1.48 - -1.33) |
| Federative Republic of Brazil | 80.01 (53.68 - 111.44) | 50.24 (34.18 - 69.53) | 87.92 (61.37 - 116.88) | 38.3 (26.62 - 51.52) | -1.21  (-1.37 - -1.05) |
| French Republic | 19.86 (13.81 - 28.03) | 34.2 (23.85 - 48.19) | 18.36 (12.63 - 25.16) | 33.43 (22.71 - 46.19) | -0.01  (-0.07 - 0.05) |
| Gabonese Republic | 0.63 (0.43 - 0.89) | 64.7 (44.58 - 88.01) | 0.81 (0.54 - 1.14) | 41.52 (28.37 - 57.42) | -1.35  (-1.4 - -1.29) |
| Georgia | 2.75 (1.89 - 3.8) | 50.64 (35.46 - 69.7) | 1.35 (0.98 - 1.77) | 44.33 (31.78 - 58.23) | -0.1  (-0.23 - 0.03) |
| Grand Duchy of Luxembourg | 0.14 (0.09 - 0.19) | 36.05 (24.76 - 50.85) | 0.2 (0.14 - 0.27) | 33.92 (23.29 - 47.17) | -0.55  (-0.68 - -0.41) |
| Greenland | 0.02 (0.02 - 0.03) | 34.71 (23.38 - 46.63) | 0.02 (0.01 - 0.02) | 32.43 (21.9 - 44.67) | -0.28  (-0.37 - -0.19) |
| Grenada | 0.04 (0.02 - 0.05) | 44.43 (30.63 - 60.14) | 0.03 (0.02 - 0.05) | 31.69 (21.57 - 43.03) | -1.01  (-1.07 - -0.95) |
| Guam | 0.11 (0.08 - 0.15) | 67.21 (47.95 - 90.37) | 0.1 (0.07 - 0.13) | 63.21 (44.89 - 84.2) | -0.27  (-0.33 - -0.22) |
| Hashemite Kingdom of Jordan | 3.3 (2.21 - 4.77) | 82.28 (58.13 - 114.38) | 7.74 (5.36 - 10.92) | 54.51 (37.89 - 76.16) | -1.35  (-1.43 - -1.28) |
| Hellenic Republic | 3.38 (2.37 - 4.67) | 33.62 (23.41 - 46.62) | 2.66 (1.87 - 3.65) | 33.07 (22.87 - 45.82) | 0.05  (0.01 - 0.09) |
| Hungary | 4.17 (2.96 - 5.59) | 42.69 (30.12 - 57.31) | 3 (2.13 - 4.07) | 37.59 (25.72 - 50.6) | -0.24  (-0.34 - -0.15) |
| Independent State of Papua New Guinea | 3.78 (2.64 - 5.24) | 91.21 (64.43 - 123.35) | 8.13 (5.68 - 11.33) | 74.37 (52.63 - 103.6) | -0.67  (-0.69 - -0.64) |
| Independent State of Samoa | 0.11 (0.08 - 0.15) | 67.44 (47.19 - 90.56) | 0.13 (0.09 - 0.17) | 61.36 (43.79 - 83.79) | -0.14  (-0.22 - -0.07) |
| Ireland | 1.33 (0.93 - 1.9) | 35.99 (25.2 - 50.63) | 1.5 (1.05 - 2.05) | 33.8 (23.24 - 46.89) | -0.13  (-0.18 - -0.09) |
| Islamic Republic of Afghanistan | 9.31 (6.16 - 13.42) | 106.47 (72.78 - 147.32) | 22.02 (14.98 - 31.48) | 68 (46.86 - 93.27) | -1.44  (-1.49 - -1.39) |
| Islamic Republic of Iran | 34.62 (23.45 - 47.95) | 62.53 (42.62 - 84.11) | 40.75 (28.04 - 54.79) | 44.9 (30.93 - 61.29) | -0.95  (-1.23 - -0.67) |
| Islamic Republic of Mauritania | 1.49 (1.01 - 2.09) | 76.31 (52.93 - 103.97) | 2.1 (1.44 - 2.98) | 47.93 (32.6 - 66.01) | -1.45  (-1.52 - -1.38) |
| Islamic Republic of Pakistan | 111.23 (77.94 - 152.65) | 105.57 (74.49 - 140.21) | 156.2 (107.31 - 211.38) | 61.21 (42.39 - 82.15) | -1.74  (-1.81 - -1.67) |
| Jamaica | 1.13 (0.75 - 1.58) | 45.54 (31.07 - 62.09) | 1.02 (0.7 - 1.42) | 32.77 (22.67 - 45.39) | -1.06  (-1.09 - -1.03) |
| Japan | 78.01 (53.29 - 106.05) | 60.25 (40.57 - 82.01) | 52.11 (37.32 - 69.32) | 52.52 (37.04 - 68.38) | -0.57  (-0.71 - -0.44) |
| Kingdom of Bahrain | 0.31 (0.21 - 0.44) | 51.88 (35.95 - 71.77) | 0.59 (0.41 - 0.8) | 32.2 (22.33 - 43.51) | -1.84  (-1.95 - -1.73) |
| Kingdom of Belgium | 3.95 (2.72 - 5.42) | 40.95 (28.26 - 56.29) | 3.72 (2.58 - 5.02) | 39.3 (27.21 - 53.89) | 0.05  (-0.15 - 0.25) |
| Kingdom of Bhutan | 0.44 (0.3 - 0.63) | 65.49 (45.24 - 89.7) | 0.35 (0.24 - 0.47) | 38.72 (26.68 - 52.74) | -1.77  (-1.85 - -1.68) |
| Kingdom of Cambodia | 9.31 (6.24 - 12.74) | 96.67 (66.13 - 129.59) | 9.95 (6.84 - 13.43) | 54.33 (38.36 - 73.65) | -1.78  (-1.91 - -1.65) |
| Kingdom of Denmark | 1.55 (1.09 - 2.17) | 29.73 (20.58 - 41.67) | 1.42 (0.97 - 1.96) | 28.51 (19.5 - 39.6) | -0.19  (-0.31 - -0.06) |
| Kingdom of Eswatini | 0.53 (0.36 - 0.74) | 68 (46.82 - 91.96) | 0.55 (0.37 - 0.77) | 42.7 (29.31 - 58.32) | -1.46  (-1.49 - -1.43) |
| Kingdom of Lesotho | 0.99 (0.67 - 1.38) | 71.47 (48.59 - 98.22) | 0.96 (0.66 - 1.36) | 44.81 (31.16 - 62.61) | -1.5  (-1.57 - -1.43) |
| Kingdom of Morocco | 19.03 (12.61 - 26.93) | 71.49 (48.38 - 98.33) | 18.56 (12.88 - 24.97) | 47.59 (32.97 - 63.91) | -1.36  (-1.39 - -1.33) |
| Kingdom of Norway | 1.33 (0.88 - 1.88) | 30.82 (20.37 - 43.49) | 1.38 (0.93 - 1.91) | 28.77 (19.36 - 39.83) | -0.2  (-0.28 - -0.11) |
| Kingdom of Saudi Arabia | 11.66 (7.8 - 16.58) | 67.29 (46.05 - 92.84) | 18.03 (12.31 - 24.44) | 37.49 (25.77 - 50.77) | -2  (-2.09 - -1.91) |
| Kingdom of Spain | 11.39 (7.99 - 15.69) | 28.84 (20.12 - 39.51) | 10.35 (7.52 - 13.66) | 27.81 (19.94 - 37.31) | -0.11  (-0.13 - -0.09) |
| Kingdom of Sweden | 2.66 (1.9 - 3.64) | 32.11 (22.54 - 44.64) | 3.26 (2.25 - 4.48) | 37.52 (25.78 - 52.18) | 0.59  (0.44 - 0.74) |
| Kingdom of Thailand | 35.32 (24.95 - 48.33) | 53.75 (38.4 - 72.67) | 28.89 (20.68 - 39.57) | 45.91 (32.02 - 61.9) | -0.43  (-0.47 - -0.4) |
| Kingdom of the Netherlands | 5.52 (3.82 - 7.64) | 34.74 (24.03 - 48.31) | 4.72 (3.29 - 6.49) | 32.87 (22.89 - 45.45) | -0.1  (-0.14 - -0.05) |
| Kingdom of Tonga | 0.08 (0.05 - 0.11) | 85.43 (60.14 - 114.51) | 0.07 (0.05 - 0.1) | 71.54 (50.6 - 97.05) | -0.5  (-0.55 - -0.45) |
| Kyrgyz Republic | 2.51 (1.69 - 3.54) | 56.47 (38.75 - 77.57) | 3.3 (2.27 - 4.49) | 47.53 (32.89 - 64.33) | -0.13  (-0.35 - 0.1) |
| Lao People's Democratic Republic | 3.49 (2.39 - 4.84) | 89.77 (62.02 - 122.59) | 4.52 (3.2 - 6.03) | 55.28 (39.57 - 73.9) | -1.65  (-1.73 - -1.56) |
| Lebanese Republic | 2.44 (1.65 - 3.51) | 80.89 (55.99 - 113.01) | 3.19 (2.24 - 4.39) | 54.74 (38.26 - 75.26) | -1.25  (-1.31 - -1.2) |
| Malaysia | 12.25 (8.41 - 17.07) | 65.51 (45.81 - 90.54) | 18.02 (12.64 - 24.91) | 50.06 (35.33 - 68.85) | -0.99  (-1.1 - -0.88) |
| Mongolia | 1.38 (0.95 - 1.94) | 61.54 (43.2 - 83.21) | 1.59 (1.1 - 2.19) | 48.65 (33.57 - 66.04) | -0.25  (-0.56 - 0.06) |
| Montenegro | 0.28 (0.2 - 0.38) | 42.91 (30.26 - 58.9) | 0.22 (0.16 - 0.29) | 38.99 (27.2 - 52.12) | -0.22  (-0.26 - -0.19) |
| New Zealand | 2.32 (1.58 - 3.28) | 63.78 (43.24 - 90.28) | 2.38 (1.68 - 3.14) | 51.34 (36.21 - 68.13) | -0.4  (-0.55 - -0.25) |
| North Macedonia | 0.93 (0.65 - 1.28) | 44.48 (31.35 - 61.35) | 0.77 (0.54 - 1.03) | 37.28 (25.75 - 49.98) | -0.51  (-0.61 - -0.4) |
| Northern Mariana Islands | 0.04 (0.03 - 0.06) | 67.66 (46.52 - 93.93) | 0.03 (0.02 - 0.03) | 54.15 (37.68 - 73.54) | -0.74  (-0.9 - -0.58) |
| Palestine | 1.99 (1.35 - 2.93) | 100.87 (69.8 - 139.48) | 3.18 (2.19 - 4.46) | 56.64 (39.6 - 76.7) | -1.96  (-2 - -1.92) |
| People's Democratic Republic of Algeria | 19.08 (12.94 - 27.57) | 73.83 (51.31 - 101.63) | 23.36 (16.11 - 31.5) | 52.47 (36.29 - 71.65) | -0.92  (-1.04 - -0.79) |
| People's Republic of Bangladesh | 76.43 (52.14 - 109.44) | 70.66 (48.33 - 97.07) | 80.78 (55.25 - 112.65) | 44.85 (30.93 - 61.72) | -1.35  (-1.45 - -1.25) |
| People's Republic of China | 666.68 (449.76 - 920.9) | 48.57 (33.46 - 67.27) | 415.09 (297.59 - 554.91) | 31.05 (22.16 - 41.44) | -1.54  (-1.7 - -1.37) |
| Plurinational State of Bolivia | 4.03 (2.76 - 5.67) | 64.15 (44.41 - 88.31) | 5.15 (3.62 - 7.18) | 40.3 (28.61 - 55.39) | -1.42  (-1.56 - -1.27) |
| Portuguese Republic | 2.81 (1.96 - 3.9) | 27.72 (19.27 - 38.42) | 2.18 (1.56 - 2.97) | 25.34 (17.7 - 34.63) | -0.24  (-0.26 - -0.22) |
| Principality of Andorra | 0.02 (0.01 - 0.03) | 30.57 (20.95 - 42.67) | 0.02 (0.02 - 0.03) | 30.42 (20.79 - 41.96) | -0.03  (-0.08 - 0.02) |
| Principality of Monaco | 0.01 (0.01 - 0.01) | 34.4 (23.98 - 46.85) | 0.01 (0.01 - 0.01) | 32.94 (22.83 - 45.23) | -0.09  (-0.12 - -0.06) |
| Puerto Rico | 1.6 (1.1 - 2.2) | 42.68 (29.18 - 58.54) | 0.89 (0.62 - 1.22) | 30.54 (20.95 - 42.16) | -1.1  (-1.14 - -1.06) |
| Republic of Albania | 1.81 (1.24 - 2.56) | 49.51 (34.18 - 67.63) | 0.99 (0.69 - 1.33) | 38.92 (27.42 - 52.69) | -0.78  (-0.86 - -0.71) |
| Republic of Angola | 8.23 (5.6 - 11.41) | 82.46 (56.22 - 111.18) | 17.19 (11.71 - 24.07) | 55.19 (38.39 - 75.9) | -1.21  (-1.32 - -1.1) |
| Republic of Armenia | 1.71 (1.19 - 2.38) | 48.82 (34.22 - 65.42) | 1.04 (0.73 - 1.4) | 37.38 (26.41 - 50.34) | -0.62  (-0.77 - -0.47) |
| Republic of Austria | 3.14 (2.21 - 4.13) | 40.24 (28.18 - 52.42) | 3.09 (2.28 - 4.09) | 41.33 (30.12 - 55.1) | 0.18  (0.06 - 0.3) |
| Republic of Azerbaijan | 4.24 (2.9 - 5.97) | 53.93 (37.23 - 74.18) | 4.42 (3.04 - 6.04) | 41.03 (28.1 - 55.62) | -0.56  (-0.69 - -0.42) |
| Republic of Belarus | 6.5 (4.6 - 8.93) | 65.06 (46.42 - 88.91) | 4.88 (3.43 - 6.49) | 62.2 (43.43 - 83.86) | 0.22  (0.04 - 0.4) |
| Republic of Benin | 3.62 (2.48 - 4.99) | 84.12 (58.15 - 113.54) | 7.37 (5.14 - 10.19) | 54.74 (38.65 - 73.63) | -1.23  (-1.31 - -1.15) |
| Republic of Botswana | 0.79 (0.54 - 1.13) | 60.92 (41.87 - 82.86) | 1.17 (0.8 - 1.6) | 42.57 (29.51 - 58.32) | -1.12  (-1.14 - -1.1) |
| Republic of Bulgaria | 3.47 (2.43 - 4.6) | 42.74 (29.78 - 57.39) | 2.03 (1.43 - 2.76) | 38.56 (27.04 - 52.77) | -0.12  (-0.21 - -0.02) |
| Republic of Burundi | 3.88 (2.67 - 5.47) | 74.9 (51.85 - 101.84) | 6.61 (4.51 - 9.48) | 49.38 (34.28 - 69.26) | -1.32  (-1.37 - -1.28) |
| Republic of Cabo Verde | 0.19 (0.13 - 0.27) | 61.07 (42.6 - 83.25) | 0.22 (0.15 - 0.31) | 34.9 (23.85 - 48.27) | -1.9  (-1.95 - -1.85) |
| Republic of Cameroon | 7.24 (4.91 - 10.22) | 74.35 (51.09 - 102.71) | 15.12 (10.05 - 20.9) | 46.19 (30.96 - 62.94) | -1.4  (-1.54 - -1.26) |
| Republic of Chad | 4.86 (3.35 - 6.82) | 91.03 (62.71 - 124.7) | 11.73 (7.77 - 16.53) | 71.57 (47.82 - 97.89) | -0.72  (-0.78 - -0.66) |
| Republic of Chile | 7.38 (5.09 - 9.89) | 51.12 (35.32 - 68.13) | 8.36 (6.06 - 10.91) | 43.53 (31.35 - 56.65) | -0.33  (-0.58 - -0.08) |
| Republic of Colombia | 16.45 (11.08 - 22.86) | 46.21 (31.66 - 62.97) | 16.26 (11.2 - 22.54) | 30.73 (21.28 - 42.14) | -1.31  (-1.34 - -1.28) |
| Republic of Costa Rica | 1.44 (0.99 - 2.01) | 44.76 (31.43 - 60.6) | 1.54 (1.06 - 2.07) | 30.82 (21.19 - 41.66) | -1.12  (-1.24 - -1) |
| Republic of Côte d'Ivoire | 8.99 (6.16 - 12.76) | 74.15 (51.39 - 101.27) | 13.98 (9.5 - 19.05) | 48.5 (33.14 - 65.37) | -1.27  (-1.31 - -1.24) |
| Republic of Croatia | 1.88 (1.32 - 2.55) | 39.34 (27.55 - 53.6) | 1.19 (0.86 - 1.58) | 34.47 (24.93 - 46.76) | -0.41  (-0.57 - -0.25) |
| Republic of Cuba | 5.31 (3.62 - 7.46) | 41.37 (28.59 - 56.71) | 3.3 (2.27 - 4.53) | 33.37 (22.55 - 46.33) | -0.53  (-0.58 - -0.47) |
| Republic of Cyprus | 0.3 (0.2 - 0.4) | 36.79 (25.35 - 50.63) | 0.42 (0.3 - 0.56) | 32.22 (22.44 - 44.71) | -0.37  (-0.43 - -0.3) |
| Republic of Djibouti | 0.31 (0.21 - 0.44) | 68.77 (47.32 - 94.49) | 0.52 (0.36 - 0.72) | 37.26 (25.82 - 50.53) | -1.92  (-2.02 - -1.82) |
| Republic of Ecuador | 5.52 (3.72 - 7.65) | 52.74 (36.65 - 71.46) | 6.83 (4.71 - 9.42) | 35.53 (24.64 - 49.07) | -1.25  (-1.31 - -1.2) |
| Republic of El Salvador | 2.58 (1.76 - 3.65) | 48.25 (33.33 - 65.27) | 2.18 (1.49 - 3.04) | 31.94 (22.06 - 44.04) | -1.38  (-1.5 - -1.25) |
| Republic of Equatorial Guinea | 0.35 (0.24 - 0.49) | 91.32 (61.95 - 125.82) | 0.67 (0.45 - 0.94) | 39.1 (26.91 - 53.81) | -2.9  (-3.03 - -2.78) |
| Republic of Estonia | 0.98 (0.7 - 1.32) | 65.65 (46.61 - 88.99) | 0.63 (0.44 - 0.84) | 59 (41.74 - 79.46) | 0.05  (-0.09 - 0.18) |
| Republic of Fiji | 0.59 (0.42 - 0.81) | 72.43 (51.25 - 98.43) | 0.58 (0.41 - 0.78) | 60.87 (42.94 - 81.84) | -0.48  (-0.54 - -0.42) |
| Republic of Finland | 1.92 (1.32 - 2.67) | 39.58 (27.21 - 55.51) | 1.61 (1.14 - 2.2) | 36.05 (25.05 - 49.83) | -0.61  (-0.81 - -0.41) |
| Republic of Ghana | 9.73 (6.73 - 13.77) | 66.64 (46.96 - 90.99) | 14.78 (10.05 - 20.37) | 40.33 (27.61 - 55.16) | -1.56  (-1.58 - -1.53) |
| Republic of Guatemala | 4.89 (3.28 - 7) | 64.42 (44.98 - 89.02) | 6.25 (4.29 - 8.73) | 35.21 (24.68 - 48.2) | -1.97  (-2.11 - -1.83) |
| Republic of Guinea | 4.57 (3.12 - 6.26) | 86.64 (59.91 - 116.81) | 7.45 (5.04 - 10.46) | 57.12 (38.96 - 77.73) | -1.24  (-1.29 - -1.2) |
| Republic of Guinea-Bissau | 0.69 (0.47 - 0.96) | 73.98 (51.88 - 100.37) | 0.97 (0.67 - 1.36) | 45.96 (31.77 - 62.85) | -1.5  (-1.54 - -1.46) |
| Republic of Guyana | 0.4 (0.27 - 0.57) | 47.21 (32.2 - 64.48) | 0.29 (0.2 - 0.4) | 35.1 (24.09 - 47.79) | -0.89  (-0.95 - -0.84) |
| Republic of Haiti | 3.91 (2.67 - 5.59) | 63.14 (44.29 - 87.92) | 5.34 (3.66 - 7.44) | 38.14 (26.27 - 52.52) | -1.61  (-1.64 - -1.58) |
| Republic of Honduras | 2.68 (1.81 - 3.81) | 60.99 (42.25 - 85.13) | 3.92 (2.66 - 5.43) | 34.51 (23.84 - 46.82) | -1.83  (-1.97 - -1.69) |
| Republic of Iceland | 0.03 (0.03 - 0.03) | 11.39 (10.34 - 12.47) | 0.07 (0.05 - 0.09) | 20.88 (14.39 - 28.97) | 2.36  (1.98 - 2.74) |
| Republic of India | 596.96 (413.8 - 809.53) | 67.78 (47.26 - 91.48) | 683.3 (471.06 - 924.45) | 42.75 (29.68 - 57.6) | -1.54  (-1.57 - -1.51) |
| Republic of Indonesia | 146.62 (101.04 - 196.82) | 74.45 (51.97 - 99.3) | 158.96 (112.92 - 213.94) | 51.57 (36.4 - 69.57) | -1.13  (-1.18 - -1.07) |
| Republic of Iraq | 17.15 (11.94 - 24.47) | 91.93 (64.87 - 126.41) | 24.23 (16.5 - 33.42) | 52.87 (36 - 73.12) | -1.93  (-2.01 - -1.85) |
| Republic of Italy | 19.53 (13.16 - 26.83) | 33.96 (22.92 - 46.69) | 14.49 (10.37 - 18.53) | 32.4 (22.85 - 41.81) | 0.16  (0.02 - 0.3) |
| Republic of Kazakhstan | 8.51 (5.81 - 11.77) | 50.55 (34.74 - 69.46) | 8.26 (5.77 - 11.21) | 46.07 (32 - 62.29) | 0.14  (-0.04 - 0.33) |
| Republic of Kenya | 15.56 (10.67 - 21.67) | 70.15 (48.13 - 94.53) | 21.38 (14.62 - 29.27) | 38.66 (26.53 - 52.18) | -1.87  (-1.89 - -1.85) |
| Republic of Kiribati | 0.07 (0.05 - 0.1) | 91.41 (64.38 - 125.75) | 0.09 (0.07 - 0.13) | 73.69 (52.76 - 99.82) | -0.67  (-0.69 - -0.65) |
| Republic of Korea | 30.1 (20.16 - 40.21) | 57.2 (38.73 - 75.54) | 22.29 (15.28 - 30.11) | 47.63 (32.81 - 64.21) | -0.54  (-0.59 - -0.49) |
| Republic of Latvia | 1.54 (1.08 - 2.1) | 60.82 (42.49 - 82.84) | 0.84 (0.6 - 1.13) | 58.01 (40.18 - 79.02) | 0.08  (-0.14 - 0.29) |
| Republic of Liberia | 1.78 (1.22 - 2.51) | 76.54 (52.54 - 104.44) | 2.46 (1.68 - 3.49) | 41.91 (28.98 - 58.25) | -2.15  (-2.23 - -2.08) |
| Republic of Lithuania | 2.59 (1.87 - 3.48) | 70.82 (50.98 - 94.77) | 1.43 (1.02 - 1.93) | 64.77 (45.99 - 88.37) | 0.05  (-0.18 - 0.29) |
| Republic of Madagascar | 7.73 (5.18 - 10.78) | 67.81 (45.53 - 92.48) | 12.16 (8.3 - 17.09) | 40.48 (27.81 - 55) | -1.65  (-1.7 - -1.6) |
| Republic of Malawi | 6.79 (4.69 - 9.67) | 70.66 (50.16 - 97.07) | 8.25 (5.64 - 11.71) | 39.7 (27.41 - 54.8) | -1.99  (-2.1 - -1.88) |
| Republic of Maldives | 0.19 (0.13 - 0.27) | 93.39 (65.27 - 127.81) | 0.25 (0.17 - 0.34) | 39.4 (27.95 - 54.53) | -2.71  (-2.79 - -2.62) |
| Republic of Mali | 6.75 (4.64 - 9.33) | 87.57 (60.27 - 119.38) | 14.51 (9.94 - 20.75) | 63.12 (43.04 - 85.8) | -1.02  (-1.06 - -0.98) |
| Republic of Malta | 0.11 (0.08 - 0.16) | 30.63 (21.25 - 43.34) | 0.1 (0.07 - 0.13) | 27 (19.08 - 37.87) | -0.29  (-0.36 - -0.22) |
| Republic of Mauritius | 0.66 (0.46 - 0.9) | 52.7 (36.92 - 71.75) | 0.58 (0.41 - 0.78) | 44.99 (32 - 61.1) | -0.54  (-0.59 - -0.5) |
| Republic of Moldova | 3.03 (2.09 - 4.13) | 70.07 (49.18 - 95.35) | 1.9 (1.31 - 2.54) | 57.75 (39.42 - 79.23) | -0.3  (-0.46 - -0.14) |
| Republic of Mozambique | 8.68 (5.9 - 11.95) | 71.58 (49.82 - 96.75) | 14.45 (9.93 - 20.33) | 46.81 (32.6 - 63.98) | -1.29  (-1.35 - -1.23) |
| Republic of Namibia | 0.92 (0.62 - 1.28) | 63.91 (44.21 - 87.01) | 1.27 (0.85 - 1.78) | 47.07 (32.1 - 64.64) | -0.94  (-1 - -0.89) |
| Republic of Nauru | 0.01 (0.01 - 0.01) | 103.06 (72.16 - 138.96) | 0.01 (0.01 - 0.01) | 71.24 (49.72 - 97.93) | -1.21  (-1.25 - -1.18) |
| Republic of Nicaragua | 2.07 (1.4 - 2.93) | 55.5 (37.9 - 77) | 2.34 (1.58 - 3.24) | 31.93 (21.83 - 44.08) | -1.61  (-1.77 - -1.44) |
| Republic of Niue | 0 (0 - 0) | 77.61 (54.31 - 104.58) | 0 (0 - 0) | 58.89 (41.35 - 79.39) | -0.86  (-0.96 - -0.77) |
| Republic of Palau | 0.01 (0.01 - 0.02) | 66.62 (47.19 - 90.1) | 0.01 (0.01 - 0.01) | 46.6 (32.33 - 63.11) | -1.05  (-1.19 - -0.92) |
| Republic of Panama | 1.06 (0.72 - 1.52) | 40.88 (28.41 - 57.25) | 1.38 (0.96 - 1.88) | 31.15 (21.66 - 42.36) | -0.81  (-0.87 - -0.76) |
| Republic of Paraguay | 2.42 (1.63 - 3.38) | 61.16 (42.05 - 83.82) | 2.76 (1.86 - 3.81) | 35.2 (24.08 - 48.41) | -1.73  (-1.78 - -1.67) |
| Republic of Peru | 11.35 (7.6 - 15.89) | 50.17 (34.44 - 68.14) | 13.06 (8.94 - 18.03) | 33.37 (22.84 - 45.89) | -1.24  (-1.29 - -1.19) |
| Republic of Poland | 14.74 (10.29 - 19.99) | 39.93 (28.07 - 54.15) | 11.88 (8.45 - 16.09) | 35.97 (25.36 - 49.04) | -0.35  (-0.53 - -0.18) |
| Republic of Rwanda | 4.79 (3.22 - 6.55) | 70.98 (49.25 - 96.3) | 5.81 (3.93 - 8.24) | 40.25 (27.59 - 54.85) | -2.05  (-2.17 - -1.92) |
| Republic of San Marino | 0.01 (0.01 - 0.01) | 34.01 (23.34 - 48.27) | 0.01 (0.01 - 0.01) | 32.8 (22.46 - 45.22) | -0.13  (-0.17 - -0.1) |
| Republic of Senegal | 5.72 (3.89 - 8.02) | 80.99 (56.63 - 109.31) | 7.85 (5.3 - 11.05) | 47.82 (32.73 - 65.21) | -1.52  (-1.59 - -1.44) |
| Republic of Serbia | 3.81 (2.67 - 5.16) | 40.48 (28.33 - 55.32) | 2.79 (1.97 - 3.81) | 34.69 (24.2 - 47.34) | -0.48  (-0.62 - -0.35) |
| Republic of Seychelles | 0.04 (0.03 - 0.06) | 56.8 (39.77 - 75.52) | 0.05 (0.04 - 0.07) | 47.29 (33.38 - 62.83) | -0.53  (-0.58 - -0.48) |
| Republic of Sierra Leone | 3.12 (2.1 - 4.29) | 76.29 (51.76 - 102.1) | 4.71 (3.21 - 6.65) | 49.15 (33.87 - 67.43) | -1.55  (-1.69 - -1.41) |
| Republic of Singapore | 2.16 (1.41 - 3.02) | 56.49 (37.72 - 77.92) | 2.59 (1.81 - 3.49) | 45.57 (30.65 - 62.3) | -0.64  (-0.73 - -0.55) |
| Republic of Slovenia | 0.95 (0.67 - 1.29) | 47.45 (33.47 - 65.18) | 0.69 (0.49 - 0.94) | 43.69 (30.31 - 60.04) | -0.04  (-0.14 - 0.07) |
| Republic of South Africa | 23.11 (15.8 - 31.51) | 57.38 (39.56 - 76.57) | 27.88 (18.94 - 37.47) | 44.66 (30.64 - 60.22) | -0.86  (-0.89 - -0.84) |
| Republic of South Sudan | 3.63 (2.42 - 5.18) | 61.67 (42.1 - 83.07) | 4.89 (3.35 - 6.84) | 52.13 (36.18 - 71.11) | -0.5  (-0.56 - -0.45) |
| Republic of Sudan | 21.13 (14.34 - 30.04) | 106.07 (73.41 - 145.61) | 28.91 (19.72 - 40.78) | 61.23 (42.34 - 85.01) | -1.87  (-1.93 - -1.81) |
| Republic of Suriname | 0.19 (0.13 - 0.27) | 46.14 (31.88 - 62.13) | 0.21 (0.15 - 0.3) | 37.04 (25.65 - 51.74) | -0.69  (-0.75 - -0.64) |
| Republic of Tajikistan | 3.66 (2.47 - 5.28) | 68.7 (47.06 - 94.08) | 4.88 (3.41 - 6.63) | 46.17 (32.5 - 62.03) | -1.02  (-1.18 - -0.86) |
| Republic of the Congo | 1.6 (1.08 - 2.26) | 66.79 (45.64 - 90.62) | 2.57 (1.82 - 3.45) | 44.32 (31.38 - 58.99) | -1.09  (-1.17 - -1) |
| Republic of the Gambia | 0.82 (0.54 - 1.16) | 84.09 (57.74 - 115.73) | 1.25 (0.85 - 1.74) | 48.96 (33.67 - 66.41) | -1.77  (-1.87 - -1.66) |
| Republic of the Marshall Islands | 0.04 (0.02 - 0.05) | 83.95 (57.94 - 113.31) | 0.04 (0.03 - 0.05) | 60.65 (43.15 - 82.25) | -0.9  (-0.96 - -0.83) |
| Republic of the Niger | 6.37 (4.34 - 9.04) | 88.37 (60.71 - 120.94) | 18.19 (12.27 - 26.2) | 77.33 (52.74 - 106.78) | -0.4  (-0.47 - -0.33) |
| Republic of the Philippines | 45.91 (31.84 - 62.2) | 70.48 (49.45 - 94.07) | 71.81 (50.11 - 96.64) | 58.46 (40.98 - 78.03) | -0.64  (-0.68 - -0.6) |
| Republic of the Union of Myanmar | 32.29 (22.18 - 44.29) | 75.33 (53.55 - 102.15) | 31.81 (22.24 - 43.47) | 53.21 (36.96 - 72.49) | -1.06  (-1.09 - -1.03) |
| Republic of Trinidad and Tobago | 0.54 (0.37 - 0.75) | 42.91 (29.47 - 58.88) | 0.44 (0.3 - 0.61) | 32.65 (22.56 - 44.31) | -0.77  (-0.85 - -0.7) |
| Republic of Tunisia | 5.99 (4.05 - 8.5) | 68.17 (46.68 - 92.99) | 5.91 (4.17 - 8.14) | 50.14 (35.2 - 69.8) | -0.92  (-1.01 - -0.82) |
| Republic of Turkey | 39.95 (27.18 - 55.81) | 65.05 (44.85 - 89.1) | 42.4 (29.59 - 57.67) | 48.47 (33.54 - 66.45) | -1.07  (-1.17 - -0.98) |
| Republic of Uganda | 12.53 (8.54 - 17.78) | 76.47 (52.33 - 104.7) | 20.05 (13.4 - 28.94) | 46.12 (31.53 - 63.83) | -1.71  (-1.8 - -1.61) |
| Republic of Uzbekistan | 12.25 (8.43 - 17.36) | 58 (40.49 - 78.64) | 15.02 (10.29 - 20.19) | 42.5 (29.11 - 57.06) | -0.75  (-0.94 - -0.57) |
| Republic of Vanuatu | 0.14 (0.1 - 0.19) | 94.49 (66.33 - 130.07) | 0.21 (0.15 - 0.29) | 66.4 (46.03 - 90.98) | -1.11  (-1.15 - -1.07) |
| Republic of Yemen | 13.89 (9.46 - 19.86) | 117.59 (82.13 - 163.69) | 21.95 (15.3 - 30.52) | 62.71 (43.48 - 86.31) | -2.08  (-2.13 - -2.04) |
| Republic of Zambia | 5.57 (3.82 - 8.13) | 71.86 (49.82 - 98.58) | 8.6 (5.88 - 12.33) | 41.58 (28.78 - 57.43) | -1.75  (-1.82 - -1.67) |
| Republic of Zimbabwe | 7.16 (4.89 - 10.21) | 70.58 (49.38 - 96.6) | 8.68 (5.92 - 12.38) | 52.78 (35.92 - 73.18) | -0.72  (-0.81 - -0.62) |
| Romania | 9.42 (6.57 - 13.04) | 41 (28.62 - 57.05) | 5.89 (4.17 - 8.04) | 38.29 (26.54 - 52.37) | -0.06  (-0.16 - 0.04) |
| Russian Federation | 99.33 (68.09 - 134.25) | 67.67 (47.5 - 91.51) | 84.21 (59.64 - 112.43) | 67.46 (47.2 - 91.04) | 0.42  (0.25 - 0.59) |
| Saint Kitts and Nevis | 0.02 (0.01 - 0.02) | 41.05 (28.42 - 55.83) | 0.02 (0.01 - 0.03) | 30.72 (20.99 - 41.88) | -0.9  (-0.93 - -0.87) |
| Saint Lucia | 0.07 (0.05 - 0.1) | 47.18 (33.21 - 64.66) | 0.06 (0.04 - 0.08) | 30.61 (21.37 - 41.58) | -1.36  (-1.42 - -1.29) |
| Saint Vincent and the Grenadines | 0.05 (0.03 - 0.07) | 43.58 (30.6 - 59.73) | 0.04 (0.03 - 0.05) | 32.59 (22.25 - 43.94) | -0.89  (-0.94 - -0.83) |
| Slovak Republic | 2.27 (1.58 - 3.01) | 43.11 (30.17 - 57.61) | 1.75 (1.23 - 2.35) | 37.1 (25.54 - 50.44) | -0.29  (-0.44 - -0.15) |
| Socialist Republic of Viet Nam | 43.74 (30.19 - 61.84) | 63.93 (45.41 - 87.93) | 49.18 (34.69 - 67.63) | 47.87 (33.73 - 65.01) | -0.78  (-0.85 - -0.72) |
| Solomon Islands | 0.37 (0.25 - 0.51) | 113.26 (79.26 - 152.22) | 0.54 (0.38 - 0.73) | 77.28 (53.87 - 102.93) | -1.21  (-1.23 - -1.19) |
| State of Eritrea | 2.21 (1.52 - 3.16) | 67.58 (47.06 - 92.79) | 2.68 (1.8 - 3.75) | 37.28 (25.39 - 51.36) | -1.97  (-2.03 - -1.9) |
| State of Israel | 2.03 (1.4 - 2.89) | 39.99 (27.71 - 56.27) | 3.63 (2.5 - 5.03) | 40.34 (27.93 - 56.33) | -0.07  (-0.12 - -0.02) |
| State of Kuwait | 1.04 (0.7 - 1.49) | 49.97 (33.99 - 71.7) | 2.52 (1.72 - 3.43) | 44.8 (30.64 - 61.39) | -0.56  (-0.69 - -0.43) |
| State of Libya | 3.54 (2.37 - 4.97) | 80.19 (55.16 - 108.66) | 3.68 (2.57 - 4.96) | 45.34 (31.5 - 61.57) | -1.78  (-2.01 - -1.56) |
| State of Qatar | 0.25 (0.17 - 0.35) | 47.93 (32.82 - 67.46) | 1.06 (0.74 - 1.47) | 29.65 (20.71 - 40.46) | -2.65  (-3.09 - -2.21) |
| Sultanate of Oman | 1.31 (0.89 - 1.84) | 65.34 (44.56 - 90.72) | 1.97 (1.34 - 2.64) | 37.2 (25.55 - 51.77) | -1.92  (-2.01 - -1.84) |
| Swiss Confederation | 2.67 (1.84 - 3.68) | 38.82 (27.06 - 53.84) | 2.77 (1.96 - 3.72) | 37.26 (25.98 - 51.59) | -0.1  (-0.17 - -0.02) |
| Syrian Arab Republic | 11.3 (7.7 - 16.33) | 90.9 (63.79 - 126.1) | 8.52 (5.83 - 11.73) | 59.07 (40.35 - 80.91) | -1.4  (-1.66 - -1.13) |
| Taiwan (Province of China) | 17.87 (12.41 - 24.23) | 78.37 (55.56 - 105.67) | 15.37 (10.97 - 21) | 64.26 (47.05 - 84.57) | -0.56  (-0.77 - -0.36) |
| Togolese Republic | 2.73 (1.87 - 3.83) | 78.8 (54.94 - 107.7) | 4.08 (2.79 - 5.57) | 47.23 (32.64 - 64.36) | -1.5  (-1.6 - -1.39) |
| Tokelau | 0 (0 - 0) | 110.97 (78.12 - 147.83) | 0 (0 - 0) | 75.2 (52.19 - 102.41) | -1.29  (-1.37 - -1.2) |
| Turkmenistan | 2.42 (1.61 - 3.43) | 63.65 (43.76 - 87.11) | 2.44 (1.69 - 3.25) | 44.92 (31.07 - 59.78) | -0.86  (-1.06 - -0.66) |
| Tuvalu | 0.01 (0.01 - 0.01) | 88.37 (62.66 - 121.5) | 0.01 (0.01 - 0.01) | 57.87 (41.11 - 78.64) | -1.35  (-1.4 - -1.31) |
| Ukraine | 34.34 (24.15 - 46.6) | 69.75 (49.64 - 95.25) | 24 (16.88 - 32.23) | 64.15 (44.86 - 86.85) | 0.02  (-0.13 - 0.17) |
| Union of the Comoros | 0.3 (0.21 - 0.43) | 68.81 (47.66 - 94.12) | 0.29 (0.2 - 0.41) | 35.94 (24.83 - 49.82) | -2.07  (-2.1 - -2.05) |
| United Arab Emirates | 1 (0.66 - 1.41) | 45.26 (30.32 - 64.91) | 2.85 (1.94 - 3.98) | 32.06 (21.68 - 45.79) | -1.52  (-2.03 - -1) |
| United Kingdom of Great Britain and Northern Ireland | 23.21 (15.91 - 31.71) | 41.11 (28.12 - 55.99) | 21.79 (15.22 - 29.21) | 38.44 (27.16 - 52.26) | -0.13  (-0.21 - -0.05) |
| United Mexican States | 47.45 (32.45 - 66.84) | 52.27 (36.22 - 70.6) | 47.28 (32.73 - 64.14) | 34.21 (23.59 - 46.54) | -1.32  (-1.41 - -1.23) |
| United Republic of Tanzania | 18.39 (12.45 - 26.23) | 74.88 (51.61 - 102.04) | 29.68 (20.18 - 41.9) | 49.31 (33.9 - 67.81) | -1.25  (-1.3 - -1.19) |
| United States of America | 122.64 (81.15 - 173.13) | 44.11 (29.64 - 62.51) | 81.02 (58.32 - 105.53) | 26.52 (18.95 - 34.77) | -2.14  (-2.37 - -1.91) |
| United States Virgin Islands | 0.05 (0.04 - 0.07) | 45.89 (32.32 - 63.36) | 0.02 (0.02 - 0.03) | 33.89 (23.55 - 46.47) | -0.98  (-1.06 - -0.91) |

ASIR, age standardized incidence rate; CI, confidence interval; EAPC, estimated annual percentage change; UI, uncertainty interval.

**Supplemental Table 2.** The DALYs and ASDR of endometriosis in 1990 and 2021, and its temporal trends from 1990 to 2021 in 204 countries and territories.

| Characteristics | 1990 | | 2021 | | 1990-2021 |
| --- | --- | --- | --- | --- | --- |
|  | Cases No.$\times$10^3^ (95% UI) | ASDR per 100,000 No. (95% UI) | Case No.$\times$10^3^ (95% UI) | ASDR per 100,000 No. (95% UI) | EAPC  No. (95% UI) |
| American Samoa | 0.03 (0.01 - 0.04) | 54.03 (31.6 - 85.76) | 0.02 (0.01 - 0.03) | 37.2 (21.53 - 59.41) | -1.19  (-1.23 - -1.16) |
| Antigua and Barbuda | 0.01 (0.01 - 0.02) | 23.64 (13.94 - 36.55) | 0.02 (0.01 - 0.03) | 18.05 (10.19 - 28.51) | -0.97 (-1.02 - -0.93) |
| Arab Republic of Egypt | 22.08 (13.09 - 34.71) | 42.44 (25.21 - 65.54) | 34.83 (19.48 - 54.86) | 32.82 (18.36 - 51.47) | -0.68 (-0.74 - -0.63) |
| Argentine Republic | 6.7 (3.87 - 10.48) | 21.16 (12.21 - 33.02) | 8.33 (4.81 - 13.14) | 17.17 (9.87 - 26.93) | -0.55  (-0.62 - -0.49) |
| Australia | 4.75 (2.65 - 7.46) | 26.1 (14.58 - 40.94) | 5.9 (3.36 - 9.46) | 23.62 (13.35 - 37.66) | -0.22  (-0.28 - -0.15) |
| Barbados | 0.06 (0.03 - 0.09) | 21.6 (12.76 - 33) | 0.05 (0.03 - 0.08) | 17.75 (10.26 - 28.37) | -0.57  (-0.64 - -0.5) |
| Belize | 0.05 (0.03 - 0.08) | 31.93 (18.58 - 48.54) | 0.09 (0.05 - 0.15) | 20.32 (11.75 - 32) | -1.3  (-1.38 - -1.23) |
| Bermuda | 0.02 (0.01 - 0.02) | 21.18 (12.17 - 32.9) | 0.01 (0.01 - 0.02) | 18.1 (10.35 - 28.76) | -0.48  (-0.57 - -0.39) |
| Bolivarian Republic of Venezuela | 5.12 (2.93 - 7.83) | 27.58 (15.95 - 42.07) | 5.22 (3.02 - 8.14) | 19.72 (11.25 - 30.38) | -1.02  (-1.13 - -0.9) |
| Bosnia and Herzegovina | 1.15 (0.65 - 1.78) | 23.12 (13.1 - 35.87) | 0.66 (0.39 - 1.03) | 21.32 (12.52 - 33.76) | -0.23  (-0.3 - -0.15) |
| Brunei Darussalam | 0.12 (0.07 - 0.19) | 40.3 (23.77 - 62.28) | 0.15 (0.09 - 0.24) | 27.57 (15.73 - 41.93) | -1.28  (-1.33 - -1.22) |
| Burkina Faso | 3.97 (2.24 - 6.32) | 55.42 (31.19 - 87.62) | 7.17 (4.12 - 10.91) | 36.84 (21.3 - 55.88) | -1.41  (-1.44 - -1.37) |
| Canada | 6.14 (3.48 - 9.74) | 19.84 (11.31 - 31.26) | 6.04 (3.39 - 9.49) | 16.79 (9.42 - 26.6) | -1.42  (-1.98 - -0.85) |
| Central African Republic | 0.99 (0.58 - 1.55) | 41.31 (24.4 - 64.61) | 1.5 (0.89 - 2.37) | 29.65 (17.62 - 46.74) | -1.02  (-1.06 - -0.98) |
| Commonwealth of Dominica | 0.02 (0.01 - 0.03) | 27.52 (15.88 - 42.55) | 0.01 (0.01 - 0.02) | 17.87 (10.2 - 28.56) | -1.34  (-1.44 - -1.24) |
| Commonwealth of the Bahamas | 0.06 (0.04 - 0.1) | 22.32 (12.91 - 35.64) | 0.08 (0.04 - 0.12) | 17.53 (10.2 - 27.5) | -0.87  (-0.92 - -0.82) |
| Cook Islands | 0.01 (0 - 0.01) | 42.31 (24.98 - 65.56) | 0.01 (0 - 0.01) | 36.06 (21.48 - 57.08) | -0.45  (-0.51 - -0.39) |
| Czech Republic | 2.69 (1.57 - 4.27) | 26.02 (15.1 - 41.32) | 2.34 (1.36 - 3.68) | 23.98 (13.9 - 37.1) | -0.07  (-0.29 - 0.16) |
| Democratic People's Republic of Korea | 12.91 (7.45 - 20.2) | 59.51 (34.2 - 93.2) | 9 (5.38 - 14.5) | 29.81 (17.66 - 47.58) | -2.2  (-2.29 - -2.11) |
| Democratic Republic of Sao Tome and Principe | 0.03 (0.02 - 0.05) | 37.73 (21.97 - 58.36) | 0.05 (0.03 - 0.07) | 20.68 (11.84 - 32.53) | -2.06  (-2.13 - -1.98) |
| Democratic Republic of the Congo | 15.57 (8.84 - 24.9) | 49.38 (28.4 - 79.05) | 23.87 (13.43 - 36.92) | 29.05 (16.5 - 44.76) | -1.61  (-1.75 - -1.47) |
| Democratic Republic of Timor-Leste | 0.46 (0.27 - 0.73) | 62.65 (36.46 - 98.09) | 0.53 (0.3 - 0.83) | 41.88 (23.53 - 66.62) | -1.54  (-1.68 - -1.4) |
| Democratic Socialist Republic of Sri Lanka | 5.29 (3.02 - 8.22) | 29.23 (16.86 - 45.65) | 5.9 (3.4 - 9.29) | 26.13 (15.08 - 41) | -0.24  (-0.29 - -0.19) |
| Dominican Republic | 2.2 (1.26 - 3.45) | 31.65 (18.22 - 48.86) | 2.62 (1.48 - 4.11) | 22.04 (12.44 - 34.37) | -1.24  (-1.32 - -1.16) |
| Eastern Republic of Uruguay | 0.73 (0.4 - 1.11) | 24.45 (13.46 - 36.98) | 0.73 (0.41 - 1.13) | 21.28 (12.14 - 33.14) | -0.45  (-0.5 - -0.4) |
| Federal Democratic Republic of Ethiopia | 18.3 (10.48 - 28.57) | 44.6 (25.82 - 69.24) | 25.82 (14.89 - 39.77) | 25.06 (14.47 - 39.09) | -1.84  (-1.88 - -1.79) |
| Federal Democratic Republic of Nepal | 9.44 (5.45 - 14.72) | 55.33 (32.2 - 86.13) | 8.94 (5.21 - 13.97) | 27.29 (15.67 - 42.78) | -2.31  (-2.45 - -2.17) |
| Federal Republic of Germany | 20.76 (11.91 - 33.43) | 24.42 (13.96 - 38.65) | 17.67 (10.09 - 28.49) | 23.2 (13.24 - 37.78) | 0.02  (-0.2 - 0.24) |
| Federal Republic of Nigeria | 35.32 (20.19 - 54.89) | 44.5 (25.53 - 68.67) | 71.4 (41 - 110.84) | 35.34 (20.38 - 54.71) | -0.68  (-0.76 - -0.6) |
| Federal Republic of Somalia | 2.99 (1.73 - 4.68) | 46.14 (26.64 - 72.05) | 6.8 (3.81 - 10.47) | 37.73 (21.03 - 57.78) | -0.64  (-0.72 - -0.57) |
| Federated States of Micronesia | 0.05 (0.03 - 0.08) | 61.13 (35.33 - 94.96) | 0.04 (0.02 - 0.06) | 35.32 (20.51 - 56.53) | -1.67  (-1.76 - -1.57) |
| Federative Republic of Brazil | 42.03 (23.77 - 67.46) | 28.15 (16.01 - 44.99) | 55.75 (32.46 - 84.36) | 23.21 (13.42 - 35.25) | -1.03  (-1.27 - -0.79) |
| French Republic | 12.34 (6.65 - 19.89) | 20.75 (11.26 - 33.08) | 11.82 (6.64 - 19.21) | 20.23 (11.09 - 32.83) | -0.02  (-0.09 - 0.06) |
| Gabonese Republic | 0.31 (0.18 - 0.47) | 35.62 (20.8 - 54.81) | 0.41 (0.24 - 0.63) | 22.65 (13.23 - 34.83) | -1.36  (-1.44 - -1.27) |
| Georgia | 1.62 (0.96 - 2.53) | 28.93 (17.14 - 44.81) | 0.85 (0.52 - 1.27) | 25.32 (15.33 - 37.96) | -0.12  (-0.23 - 0) |
| Grand Duchy of Luxembourg | 0.09 (0.05 - 0.15) | 22.52 (12.57 - 35.61) | 0.14 (0.07 - 0.22) | 20.61 (11.19 - 33.53) | -0.84  (-1.05 - -0.63) |
| Greenland | 0.01 (0.01 - 0.02) | 19.02 (10.53 - 29.72) | 0.01 (0.01 - 0.02) | 18.04 (10.4 - 28.61) | -0.27  (-0.35 - -0.19) |
| Grenada | 0.02 (0.01 - 0.03) | 25.73 (14.79 - 39.74) | 0.02 (0.01 - 0.03) | 18.21 (10.53 - 28.69) | -0.99  (-1.08 - -0.89) |
| Guam | 0.06 (0.03 - 0.09) | 38.45 (22.24 - 60.42) | 0.06 (0.03 - 0.09) | 37.36 (21.89 - 58) | -0.14  (-0.22 - -0.07) |
| Hashemite Kingdom of Jordan | 1.68 (0.95 - 2.59) | 50.65 (29.69 - 77.79) | 4.5 (2.54 - 6.92) | 33.16 (18.62 - 51.39) | -1.44  (-1.5 - -1.38) |
| Hellenic Republic | 2.09 (1.2 - 3.35) | 20.2 (11.5 - 32.3) | 1.8 (1.01 - 2.94) | 19.76 (11.35 - 32.44) | 0.04  (-0.01 - 0.09) |
| Hungary | 2.56 (1.47 - 3.93) | 24.98 (14.2 - 38.19) | 1.98 (1.14 - 3.11) | 21.96 (12.37 - 34.63) | -0.24  (-0.34 - -0.14) |
| Independent State of Papua New Guinea | 2.09 (1.19 - 3.34) | 55.82 (32.24 - 88.85) | 4.87 (2.72 - 7.77) | 46.77 (26.46 - 74.48) | -0.59  (-0.63 - -0.55) |
| Independent State of Samoa | 0.05 (0.03 - 0.08) | 37.88 (22.06 - 59.09) | 0.07 (0.04 - 0.1) | 35.25 (21.05 - 54.35) | -0.01  (-0.1 - 0.09) |
| Ireland | 0.8 (0.46 - 1.27) | 22.53 (13.02 - 36.21) | 0.96 (0.54 - 1.53) | 20.17 (11.14 - 32.3) | -0.24  (-0.3 - -0.18) |
| Islamic Republic of Afghanistan | 4.72 (2.73 - 7.29) | 68.95 (39.78 - 108.82) | 12.69 (7.11 - 19.57) | 45.9 (25.26 - 71.85) | -1.34  (-1.52 - -1.16) |
| Islamic Republic of Iran | 17.31 (9.8 - 26.76) | 35.54 (20.59 - 54.05) | 25.57 (14.62 - 39.66) | 25.77 (14.95 - 39.25) | -0.93  (-1.22 - -0.63) |
| Islamic Republic of Mauritania | 0.78 (0.45 - 1.23) | 44.96 (26.25 - 70.68) | 1.09 (0.62 - 1.67) | 27.85 (16.19 - 43.56) | -1.47  (-1.55 - -1.39) |
| Islamic Republic of Pakistan | 61.87 (36.44 - 96.19) | 67.2 (39.33 - 104.49) | 89.15 (51.57 - 137.42) | 37.29 (21.64 - 56.81) | -1.92  (-2.01 - -1.83) |
| Jamaica | 0.59 (0.34 - 0.9) | 26.29 (15.33 - 40.1) | 0.6 (0.34 - 0.94) | 18.99 (10.87 - 30.04) | -1.03  (-1.06 - -1) |
| Japan | 46.4 (26.82 - 72.55) | 34.62 (19.63 - 54.16) | 33.74 (20.7 - 50.63) | 29.78 (18.3 - 44.06) | -0.66  (-0.81 - -0.51) |
| Kingdom of Bahrain | 0.17 (0.09 - 0.27) | 26.39 (15.43 - 41.85) | 0.34 (0.19 - 0.53) | 16.67 (9.49 - 25.76) | -1.85  (-1.99 - -1.71) |
| Kingdom of Belgium | 2.82 (1.58 - 4.54) | 27.37 (15.47 - 43.63) | 2.69 (1.48 - 4.29) | 25.75 (14.01 - 41.47) | 0.08  (-0.27 - 0.43) |
| Kingdom of Bhutan | 0.23 (0.13 - 0.36) | 40.14 (23.13 - 63.93) | 0.2 (0.11 - 0.31) | 22.16 (12.59 - 34.47) | -2.01  (-2.11 - -1.91) |
| Kingdom of Cambodia | 5.3 (3.07 - 8.22) | 61.8 (36.28 - 96.19) | 5.61 (3.31 - 8.87) | 31.08 (18.31 - 48.92) | -2.13  (-2.32 - -1.95) |
| Kingdom of Denmark | 0.92 (0.52 - 1.49) | 16.88 (9.55 - 27.51) | 0.86 (0.49 - 1.37) | 15.98 (9.06 - 25.88) | -0.22  (-0.35 - -0.08) |
| Kingdom of Eswatini | 0.27 (0.16 - 0.43) | 41.29 (24.36 - 65.67) | 0.29 (0.17 - 0.46) | 24.23 (13.96 - 37.96) | -1.7  (-1.73 - -1.68) |
| Kingdom of Lesotho | 0.55 (0.31 - 0.86) | 44.26 (25.11 - 69.44) | 0.51 (0.29 - 0.81) | 26.01 (15.09 - 41.44) | -1.72  (-1.83 - -1.62) |
| Kingdom of Morocco | 9.7 (5.52 - 15.42) | 40.1 (22.84 - 63.15) | 10.57 (6.11 - 16.08) | 26.8 (15.53 - 40.77) | -1.36  (-1.44 - -1.29) |
| Kingdom of Norway | 0.77 (0.41 - 1.25) | 17.38 (9.28 - 28.28) | 0.86 (0.49 - 1.36) | 16.46 (9.29 - 25.86) | -0.11  (-0.21 - -0.01) |
| Kingdom of Saudi Arabia | 6.04 (3.37 - 9.52) | 38.21 (21.64 - 59.72) | 10.59 (6.24 - 15.94) | 20.16 (11.76 - 30.33) | -2.17  (-2.27 - -2.08) |
| Kingdom of Spain | 6.42 (3.66 - 10.06) | 16.37 (9.29 - 25.63) | 6.53 (3.89 - 9.93) | 15.45 (9.14 - 23.33) | -0.15  (-0.18 - -0.12) |
| Kingdom of Sweden | 1.64 (0.95 - 2.62) | 18.78 (10.66 - 29.9) | 2.05 (1.2 - 3.23) | 21.58 (12.61 - 33.7) | 0.54  (0.38 - 0.7) |
| Kingdom of Thailand | 17.96 (10.48 - 27.31) | 28.8 (16.77 - 43.78) | 17.37 (10.06 - 27.31) | 25.1 (14.46 - 39.97) | -0.39  (-0.43 - -0.34) |
| Kingdom of the Netherlands | 3.54 (2.01 - 5.68) | 21.33 (12.06 - 34.59) | 3.05 (1.76 - 4.89) | 19.61 (11.2 - 31.49) | -0.17  (-0.23 - -0.11) |
| Kingdom of Tonga | 0.04 (0.02 - 0.06) | 51.21 (30.09 - 79.7) | 0.04 (0.02 - 0.06) | 43.37 (25.5 - 68.58) | -0.44  (-0.48 - -0.4) |
| Kyrgyz Republic | 1.4 (0.81 - 2.22) | 33.48 (19.1 - 52.21) | 2 (1.16 - 3.16) | 28.42 (16.49 - 44.82) | -0.07  (-0.31 - 0.18) |
| Lao People's Democratic Republic | 1.95 (1.13 - 3) | 55.62 (32 - 86.74) | 2.53 (1.45 - 3.92) | 31.76 (18.27 - 49.12) | -1.96  (-2.08 - -1.84) |
| Lebanese Republic | 1.36 (0.77 - 2.15) | 48.03 (27.45 - 75.69) | 2.11 (1.21 - 3.31) | 33.4 (19.37 - 51.99) | -1.13  (-1.17 - -1.08) |
| Malaysia | 6.41 (3.67 - 10.11) | 36.43 (21.05 - 56.57) | 10.15 (5.89 - 15.84) | 27.96 (16.31 - 43.72) | -1.01  (-1.13 - -0.88) |
| Mongolia | 0.75 (0.43 - 1.17) | 37.38 (21.91 - 58.28) | 1.01 (0.57 - 1.59) | 28.99 (16.45 - 45.22) | -0.2  (-0.55 - 0.15) |
| Montenegro | 0.16 (0.09 - 0.25) | 25.17 (14.51 - 38.67) | 0.14 (0.08 - 0.22) | 23.24 (13.8 - 35.99) | -0.18  (-0.21 - -0.16) |
| New Zealand | 1.41 (0.81 - 2.27) | 38.77 (22.27 - 62.24) | 1.51 (0.94 - 2.23) | 30 (18.69 - 43.87) | -0.47  (-0.68 - -0.26) |
| North Macedonia | 0.55 (0.33 - 0.86) | 26.31 (15.65 - 41.02) | 0.5 (0.3 - 0.78) | 21.67 (12.67 - 33.56) | -0.58  (-0.69 - -0.48) |
| Northern Mariana Islands | 0.02 (0.01 - 0.03) | 35.35 (20.53 - 55.66) | 0.02 (0.01 - 0.02) | 30.15 (17.74 - 48.16) | -0.53  (-0.67 - -0.39) |
| Palestine | 1.12 (0.64 - 1.75) | 68.47 (39 - 105.99) | 1.8 (0.99 - 2.84) | 34.66 (19.39 - 54.23) | -2.46  (-2.6 - -2.32) |
| People's Democratic Republic of Algeria | 9.51 (5.37 - 14.55) | 42.2 (24.36 - 64.24) | 14.4 (8.33 - 22.16) | 30.79 (17.78 - 47.6) | -0.77  (-0.9 - -0.65) |
| People's Republic of Bangladesh | 40.28 (22.67 - 63.45) | 42.35 (23.85 - 68.25) | 45.91 (25.98 - 70.5) | 25.98 (14.7 - 39.74) | -1.39  (-1.51 - -1.27) |
| People's Republic of China | 348.78 (193.76 - 569.25) | 27.26 (15.37 - 45.03) | 269.43 (157.66 - 426.16) | 17.76 (10.61 - 28.04) | -1.48  (-1.64 - -1.32) |
| Plurinational State of Bolivia | 2.22 (1.29 - 3.46) | 39.22 (22.99 - 60.85) | 3 (1.69 - 4.67) | 23.89 (13.5 - 37.01) | -1.44  (-1.62 - -1.25) |
| Portuguese Republic | 1.57 (0.89 - 2.54) | 15.46 (8.83 - 25.11) | 1.32 (0.75 - 2.05) | 13.78 (8.04 - 21.64) | -0.3  (-0.33 - -0.27) |
| Principality of Andorra | 0.01 (0.01 - 0.02) | 17.87 (10.33 - 28.39) | 0.02 (0.01 - 0.02) | 17.58 (9.74 - 28.24) | -0.04  (-0.11 - 0.03) |
| Principality of Monaco | 0.01 (0 - 0.01) | 20.98 (11.85 - 33.57) | 0.01 (0 - 0.01) | 19.53 (11.1 - 30.26) | -0.17  (-0.2 - -0.13) |
| Puerto Rico | 0.89 (0.51 - 1.45) | 24.17 (13.98 - 39.62) | 0.53 (0.3 - 0.85) | 17.16 (9.59 - 27.42) | -1.13  (-1.19 - -1.07) |
| Republic of Albania | 1.05 (0.6 - 1.63) | 30.19 (17.32 - 47.61) | 0.62 (0.38 - 1) | 23.31 (14.12 - 37.15) | -0.88  (-0.96 - -0.8) |
| Republic of Angola | 4.52 (2.61 - 6.91) | 50.94 (29.45 - 78.67) | 9.52 (5.51 - 14.67) | 34.58 (19.89 - 53.48) | -1.13  (-1.25 - -1.01) |
| Republic of Armenia | 0.99 (0.56 - 1.55) | 27.69 (15.96 - 42.65) | 0.63 (0.38 - 0.97) | 20.59 (12.36 - 31.16) | -0.72  (-0.87 - -0.57) |
| Republic of Austria | 2.18 (1.35 - 3.24) | 26.17 (16.17 - 38.95) | 2.39 (1.44 - 3.64) | 28.02 (16.8 - 42.73) | 0.36  (0.19 - 0.53) |
| Republic of Azerbaijan | 2.37 (1.38 - 3.7) | 31.31 (18.15 - 49.4) | 2.77 (1.65 - 4.31) | 23.47 (13.89 - 36.02) | -0.58  (-0.71 - -0.44) |
| Republic of Belarus | 4.19 (2.33 - 6.67) | 39.32 (21.97 - 62.29) | 3.44 (1.97 - 5.29) | 37.86 (21.94 - 58.92) | 0.24  (0.07 - 0.41) |
| Republic of Benin | 1.94 (1.12 - 3.05) | 50.58 (28.94 - 78.13) | 3.99 (2.34 - 6.21) | 33.7 (19.88 - 52.44) | -1.04  (-1.15 - -0.93) |
| Republic of Botswana | 0.4 (0.23 - 0.61) | 35.33 (20.42 - 54.28) | 0.67 (0.4 - 1.03) | 24.25 (14.65 - 37.42) | -1.14  (-1.18 - -1.11) |
| Republic of Bulgaria | 2.09 (1.22 - 3.28) | 24.74 (14.31 - 38.24) | 1.39 (0.81 - 2.15) | 22.64 (12.75 - 35.1) | -0.04  (-0.13 - 0.05) |
| Republic of Burundi | 2.12 (1.2 - 3.36) | 45.64 (25.87 - 71.49) | 3.79 (2.08 - 5.98) | 31.99 (17.88 - 50.01) | -1.12  (-1.15 - -1.08) |
| Republic of Cabo Verde | 0.09 (0.05 - 0.15) | 34.19 (19.72 - 53.68) | 0.12 (0.07 - 0.19) | 18.75 (10.85 - 29.09) | -2.05  (-2.11 - -1.99) |
| Republic of Cameroon | 3.68 (2.1 - 5.75) | 42.57 (24.11 - 66.64) | 7.84 (4.56 - 12.12) | 26.57 (15.77 - 41.11) | -1.32  (-1.48 - -1.16) |
| Republic of Chad | 2.67 (1.47 - 4.29) | 57.51 (32.61 - 91.53) | 6.99 (3.81 - 10.66) | 51.78 (28.4 - 78.85) | -0.27  (-0.3 - -0.24) |
| Republic of Chile | 4.29 (2.46 - 6.72) | 31.06 (18.06 - 49.28) | 5.51 (3.41 - 8.1) | 27.19 (16.73 - 40.28) | -0.16  (-0.54 - 0.21) |
| Republic of Colombia | 8.6 (4.88 - 13.71) | 25.93 (14.93 - 40.69) | 9.55 (5.64 - 15.04) | 17.74 (10.5 - 27.94) | -1.22  (-1.24 - -1.2) |
| Republic of Costa Rica | 0.77 (0.44 - 1.18) | 25.19 (14.74 - 38.7) | 0.91 (0.52 - 1.37) | 17.48 (10.11 - 26.36) | -1.09  (-1.21 - -0.96) |
| Republic of Côte d'Ivoire | 4.65 (2.64 - 7.41) | 42.89 (24.66 - 66.69) | 7.7 (4.37 - 11.81) | 28.87 (16.64 - 44.05) | -1.17  (-1.21 - -1.14) |
| Republic of Croatia | 1.13 (0.65 - 1.82) | 22.24 (12.82 - 35.79) | 0.72 (0.46 - 1.1) | 18.95 (12.12 - 28.71) | -0.58  (-0.76 - -0.39) |
| Republic of Cuba | 2.84 (1.63 - 4.43) | 23.15 (13.43 - 35.51) | 2.18 (1.27 - 3.43) | 19.57 (11.61 - 30.57) | -0.33  (-0.41 - -0.26) |
| Republic of Cyprus | 0.19 (0.11 - 0.31) | 23.6 (13.03 - 37.63) | 0.29 (0.16 - 0.45) | 19.37 (10.99 - 30.48) | -0.55  (-0.65 - -0.45) |
| Republic of Djibouti | 0.16 (0.09 - 0.26) | 41.98 (24.19 - 65.48) | 0.31 (0.18 - 0.5) | 22.69 (13.16 - 36.07) | -1.91  (-2.01 - -1.81) |
| Republic of Ecuador | 2.82 (1.59 - 4.39) | 29.8 (16.73 - 45.27) | 3.83 (2.23 - 5.98) | 20.19 (11.8 - 31.52) | -1.21  (-1.26 - -1.16) |
| Republic of El Salvador | 1.3 (0.75 - 2.06) | 27.4 (15.77 - 42.52) | 1.24 (0.7 - 1.95) | 18.47 (10.55 - 28.66) | -1.3  (-1.44 - -1.15) |
| Republic of Equatorial Guinea | 0.19 (0.11 - 0.3) | 57.07 (32.14 - 89.46) | 0.35 (0.2 - 0.54) | 23.08 (13.4 - 35.6) | -3.13  (-3.26 - -2.99) |
| Republic of Estonia | 0.64 (0.38 - 1.02) | 40.56 (23.6 - 64.97) | 0.43 (0.25 - 0.68) | 35.25 (20.39 - 55.57) | -0.04  (-0.18 - 0.09) |
| Republic of Fiji | 0.31 (0.18 - 0.48) | 40.47 (23.36 - 62.93) | 0.33 (0.19 - 0.51) | 34.76 (20.23 - 53.9) | -0.39  (-0.46 - -0.32) |
| Republic of Finland | 1.39 (0.77 - 2.22) | 26.09 (14.42 - 40.95) | 1.1 (0.61 - 1.77) | 22.61 (12.56 - 35.78) | -0.96  (-1.22 - -0.7) |
| Republic of Ghana | 4.9 (2.83 - 7.65) | 37.26 (21.89 - 58.06) | 7.66 (4.36 - 11.72) | 22.25 (12.69 - 34.01) | -1.57  (-1.6 - -1.53) |
| Republic of Guatemala | 2.77 (1.59 - 4.34) | 41.76 (23.9 - 65.32) | 3.44 (1.97 - 5.33) | 20.89 (12.21 - 32.18) | -2.26  (-2.45 - -2.07) |
| Republic of Guinea | 2.57 (1.45 - 4.02) | 52.79 (30.09 - 82.42) | 4.08 (2.31 - 6.57) | 35.2 (20.15 - 56.97) | -1.23  (-1.27 - -1.19) |
| Republic of Guinea-Bissau | 0.35 (0.2 - 0.55) | 42.6 (24.57 - 67.05) | 0.5 (0.29 - 0.78) | 26.31 (15.31 - 40.38) | -1.52  (-1.55 - -1.5) |
| Republic of Guyana | 0.21 (0.12 - 0.33) | 27.22 (15.42 - 41.93) | 0.16 (0.1 - 0.25) | 20.29 (11.73 - 31.01) | -0.83  (-0.88 - -0.77) |
| Republic of Haiti | 2.32 (1.34 - 3.56) | 41.21 (23.91 - 63.04) | 3.08 (1.77 - 4.83) | 22.84 (13.26 - 35.85) | -1.89  (-1.92 - -1.87) |
| Republic of Honduras | 1.48 (0.82 - 2.36) | 38.49 (21.72 - 60.19) | 2.17 (1.24 - 3.4) | 20.48 (11.74 - 31.96) | -2.05  (-2.23 - -1.87) |
| Republic of Iceland | 0.03 (0.02 - 0.04) | 9.74 (5.77 - 14.85) | 0.04 (0.02 - 0.07) | 12.6 (6.83 - 19.55) | 1.16  (0.89 - 1.44) |
| Republic of India | 337.54 (193.23 - 518.06) | 41.31 (23.75 - 64.09) | 392.97 (227.62 - 603.47) | 25.11 (14.63 - 38.37) | -1.68  (-1.71 - -1.64) |
| Republic of Indonesia | 78.53 (44.94 - 121.33) | 43.13 (24.84 - 67.06) | 95.5 (56.26 - 149.48) | 30.22 (17.77 - 47.06) | -1.07  (-1.13 - -1.01) |
| Republic of Iraq | 9.46 (5.34 - 14.84) | 59.56 (34.17 - 92.27) | 13.67 (7.72 - 21.85) | 31.21 (17.54 - 49.43) | -2.33  (-2.41 - -2.25) |
| Republic of Italy | 12.79 (7.26 - 19.89) | 21.61 (12.17 - 33.81) | 10.25 (6.44 - 14.73) | 19.99 (12.66 - 28.73) | -0.14  (-0.21 - -0.08) |
| Republic of Kazakhstan | 4.88 (2.74 - 7.71) | 28.86 (16.27 - 45.18) | 5.28 (3.01 - 8.16) | 27.26 (15.65 - 41.86) | 0.28  (0.09 - 0.47) |
| Republic of Kenya | 7.74 (4.41 - 12.01) | 41.37 (23.99 - 63.71) | 11.15 (6.32 - 17.43) | 22.45 (12.8 - 34.75) | -1.91  (-1.93 - -1.89) |
| Republic of Kiribati | 0.04 (0.02 - 0.06) | 55.35 (32.15 - 85.33) | 0.05 (0.03 - 0.08) | 44.98 (25.49 - 70.15) | -0.61  (-0.64 - -0.58) |
| Republic of Korea | 17.08 (9.68 - 27.11) | 33.37 (19.11 - 52.13) | 14.88 (8.84 - 23.32) | 27.32 (16.42 - 41.96) | -0.63  (-0.67 - -0.58) |
| Republic of Latvia | 0.98 (0.56 - 1.54) | 36.36 (20.51 - 56.49) | 0.58 (0.34 - 0.9) | 34.31 (20.14 - 53.38) | -0.04  (-0.32 - 0.24) |
| Republic of Liberia | 0.93 (0.52 - 1.46) | 44.17 (25.06 - 68.58) | 1.22 (0.69 - 1.88) | 22.82 (13.01 - 35.11) | -2.33  (-2.4 - -2.25) |
| Republic of Lithuania | 1.72 (1.03 - 2.74) | 45.25 (26.99 - 71.62) | 1.01 (0.6 - 1.58) | 40.41 (23.82 - 63.2) | -0.19  (-0.42 - 0.05) |
| Republic of Madagascar | 3.97 (2.18 - 6.21) | 39.81 (21.95 - 62.96) | 6.44 (3.68 - 10.35) | 24.05 (13.9 - 38.67) | -1.62  (-1.67 - -1.57) |
| Republic of Malawi | 3.47 (1.99 - 5.56) | 41.58 (23.85 - 65.8) | 4.1 (2.31 - 6.41) | 22.98 (13.14 - 35.93) | -2.06  (-2.19 - -1.94) |
| Republic of Maldives | 0.11 (0.06 - 0.16) | 59.71 (34.31 - 92.12) | 0.15 (0.08 - 0.23) | 20.98 (12.2 - 33.37) | -3.17  (-3.29 - -3.05) |
| Republic of Mali | 3.75 (2.15 - 5.88) | 54.15 (31.21 - 85.48) | 8.11 (4.54 - 12.6) | 41.87 (23.66 - 65.03) | -0.79  (-0.82 - -0.77) |
| Republic of Malta | 0.07 (0.04 - 0.11) | 17.87 (10.2 - 27.65) | 0.06 (0.03 - 0.1) | 15 (8.57 - 23.84) | -0.41  (-0.5 - -0.31) |
| Republic of Mauritius | 0.34 (0.2 - 0.53) | 28.27 (16.53 - 43.2) | 0.33 (0.19 - 0.51) | 24.21 (14.19 - 37.2) | -0.53  (-0.58 - -0.48) |
| Republic of Moldova | 1.99 (1.14 - 3.14) | 43.86 (25.42 - 69.05) | 1.3 (0.74 - 2.05) | 34.08 (19.57 - 54.27) | -0.51  (-0.67 - -0.36) |
| Republic of Mozambique | 4.51 (2.57 - 7.18) | 41.36 (23.75 - 65.93) | 7.53 (4.19 - 11.88) | 28.41 (15.92 - 45) | -1.07  (-1.15 - -0.99) |
| Republic of Namibia | 0.48 (0.28 - 0.76) | 38.65 (22.12 - 59.37) | 0.71 (0.4 - 1.14) | 27.83 (15.74 - 44.36) | -0.98  (-1.05 - -0.91) |
| Republic of Nauru | 0.01 (0 - 0.01) | 67.07 (39.05 - 104.04) | 0 (0 - 0.01) | 43.64 (25.52 - 68.9) | -1.46  (-1.5 - -1.42) |
| Republic of Nicaragua | 1.07 (0.6 - 1.65) | 33.15 (18.85 - 50.22) | 1.33 (0.74 - 2.07) | 18.48 (10.38 - 29.07) | -1.64  (-1.85 - -1.44) |
| Republic of Niue | 0 (0 - 0) | 45.72 (27.15 - 71.29) | 0 (0 - 0) | 34.04 (19.82 - 52.96) | -0.94  (-1.05 - -0.84) |
| Republic of Palau | 0.01 (0 - 0.01) | 36.9 (21.67 - 57.16) | 0.01 (0 - 0.01) | 25.28 (15.37 - 39.51) | -1.06  (-1.21 - -0.91) |
| Republic of Panama | 0.54 (0.32 - 0.84) | 22.62 (13.21 - 34.49) | 0.8 (0.45 - 1.24) | 18.06 (10.18 - 28.03) | -0.62  (-0.68 - -0.56) |
| Republic of Paraguay | 1.36 (0.77 - 2.13) | 37.31 (21.3 - 58.37) | 1.56 (0.89 - 2.45) | 20.3 (11.7 - 31.64) | -1.87  (-1.93 - -1.8) |
| Republic of Peru | 5.61 (3.05 - 8.69) | 27.41 (15.05 - 42.25) | 7.46 (4.34 - 11.56) | 18.9 (10.98 - 29.23) | -1.1  (-1.15 - -1.04) |
| Republic of Poland | 9.15 (5.36 - 14.16) | 23.57 (13.78 - 36.27) | 8.04 (4.77 - 12.61) | 21.27 (12.39 - 32.94) | -0.34  (-0.52 - -0.16) |
| Republic of Rwanda | 2.5 (1.41 - 3.94) | 42.23 (24.15 - 65.46) | 3.11 (1.75 - 4.9) | 23.75 (13.36 - 37.12) | -2.1  (-2.22 - -1.98) |
| Republic of San Marino | 0.01 (0 - 0.01) | 20.27 (11.27 - 31.92) | 0.01 (0 - 0.01) | 19.51 (11.01 - 30.38) | -0.11  (-0.15 - -0.06) |
| Republic of Senegal | 2.97 (1.69 - 4.71) | 48.4 (27.79 - 76.21) | 4.16 (2.33 - 6.61) | 28.34 (16.01 - 44.57) | -1.47  (-1.58 - -1.37) |
| Republic of Serbia | 2.28 (1.34 - 3.53) | 23.25 (13.59 - 35.99) | 1.73 (1.02 - 2.69) | 19.73 (11.56 - 30.78) | -0.58  (-0.75 - -0.41) |
| Republic of Seychelles | 0.02 (0.01 - 0.03) | 30.61 (18.1 - 47.37) | 0.03 (0.02 - 0.05) | 26.16 (15.35 - 41.99) | -0.41  (-0.46 - -0.36) |
| Republic of Sierra Leone | 1.67 (0.9 - 2.56) | 44.72 (24.75 - 68.82) | 2.45 (1.37 - 3.77) | 28.54 (15.87 - 44.5) | -1.55  (-1.7 - -1.4) |
| Republic of Singapore | 1.26 (0.73 - 1.98) | 32.88 (19.16 - 51.36) | 1.69 (0.97 - 2.64) | 25.8 (14.68 - 40.32) | -0.82  (-0.95 - -0.7) |
| Republic of Slovenia | 0.6 (0.35 - 0.93) | 28.55 (16.68 - 44.34) | 0.5 (0.29 - 0.79) | 27.23 (15.37 - 42.87) | 0.06  (-0.04 - 0.17) |
| Republic of South Africa | 12.19 (7.12 - 18.59) | 33.32 (19.55 - 51) | 16.44 (9.42 - 25.46) | 25.76 (14.79 - 39.66) | -0.88  (-0.9 - -0.85) |
| Republic of South Sudan | 1.79 (1 - 2.8) | 35.2 (20.2 - 55.28) | 2.76 (1.55 - 4.24) | 34.68 (19.37 - 53.8) | 0.02  (-0.01 - 0.06) |
| Republic of Sudan | 12.37 (6.99 - 19.44) | 71.88 (41.13 - 111.9) | 16.5 (9.47 - 25.78) | 38.53 (21.98 - 59.14) | -2.23  (-2.4 - -2.06) |
| Republic of Suriname | 0.1 (0.06 - 0.16) | 27.27 (15.88 - 41.97) | 0.13 (0.08 - 0.21) | 22.21 (12.8 - 34.99) | -0.63  (-0.7 - -0.57) |
| Republic of Tajikistan | 2.09 (1.19 - 3.34) | 44.17 (25.9 - 70.21) | 2.84 (1.62 - 4.33) | 27.15 (15.67 - 41.22) | -1.32  (-1.47 - -1.17) |
| Republic of the Congo | 0.77 (0.44 - 1.19) | 37.26 (21.58 - 57.12) | 1.38 (0.78 - 2.09) | 25.08 (14.27 - 38.1) | -0.97  (-1.08 - -0.87) |
| Republic of the Gambia | 0.44 (0.25 - 0.7) | 51.3 (30.23 - 81.05) | 0.64 (0.37 - 1.02) | 28.35 (16.54 - 45.23) | -1.96  (-2.07 - -1.85) |
| Republic of the Marshall Islands | 0.02 (0.01 - 0.03) | 49.5 (27.85 - 78.98) | 0.02 (0.01 - 0.03) | 34.66 (19.76 - 53.62) | -0.95  (-1.04 - -0.86) |
| Republic of the Niger | 3.46 (1.95 - 5.46) | 54.59 (31.12 - 85.02) | 11.35 (6.31 - 17.64) | 61.45 (34.29 - 95.47) | 0.46  (0.42 - 0.51) |
| Republic of the Philippines | 23.86 (13.64 - 36.89) | 40.56 (23.69 - 62.73) | 40.23 (23.38 - 62) | 33.75 (19.65 - 52) | -0.57  (-0.62 - -0.53) |
| Republic of the Union of Myanmar | 17.07 (9.68 - 26.67) | 43.64 (25.35 - 67.95) | 17.6 (10.57 - 27.38) | 29.85 (17.86 - 46.57) | -1.15  (-1.18 - -1.11) |
| Republic of Trinidad and Tobago | 0.3 (0.17 - 0.47) | 24.44 (13.98 - 38.19) | 0.27 (0.15 - 0.41) | 18.67 (10.65 - 28.31) | -0.71  (-0.8 - -0.62) |
| Republic of Tunisia | 3.06 (1.81 - 4.63) | 38.21 (22.65 - 58.42) | 3.62 (2.12 - 5.61) | 28.88 (16.83 - 44.59) | -0.82  (-0.9 - -0.74) |
| Republic of Turkey | 20.38 (11.76 - 32.13) | 36.05 (20.85 - 57.57) | 25.13 (14.43 - 39.31) | 27.78 (15.81 - 43.46) | -1  (-1.08 - -0.92) |
| Republic of Uganda | 6.43 (3.63 - 10.25) | 46.39 (27.05 - 72.6) | 10.68 (6.02 - 16.76) | 28.94 (16.11 - 44.77) | -1.65  (-1.77 - -1.53) |
| Republic of Uzbekistan | 6.67 (3.67 - 10.31) | 34.4 (19.43 - 52.99) | 8.97 (5.37 - 14.5) | 24.31 (14.63 - 39.2) | -0.88  (-1.07 - -0.69) |
| Republic of Vanuatu | 0.08 (0.04 - 0.12) | 57.86 (32.85 - 92.59) | 0.12 (0.07 - 0.19) | 39.17 (22.66 - 62.2) | -1.29  (-1.33 - -1.25) |
| Republic of Yemen | 9.02 (5.13 - 14.17) | 88.27 (50.16 - 138.51) | 12.57 (7.34 - 19.03) | 39.63 (23.43 - 60.44) | -2.85  (-3.03 - -2.67) |
| Republic of Zambia | 2.73 (1.57 - 4.25) | 42.32 (24.22 - 66.96) | 4.53 (2.65 - 7.4) | 24.64 (14.42 - 39.93) | -1.72  (-1.8 - -1.63) |
| Republic of Zimbabwe | 3.75 (2.16 - 5.81) | 44.18 (25.29 - 68.01) | 4.87 (2.81 - 7.87) | 32.93 (19.18 - 53.44) | -0.64  (-0.78 - -0.51) |
| Romania | 5.34 (3.17 - 8.28) | 23.32 (13.64 - 36.14) | 3.9 (2.26 - 6.2) | 22.46 (12.91 - 35.41) | 0.04  (-0.05 - 0.14) |
| Russian Federation | 64.19 (36.81 - 99.95) | 40.65 (23.62 - 63.15) | 57.97 (33.37 - 90.83) | 40.75 (23.69 - 62.99) | 0.46  (0.29 - 0.64) |
| Saint Kitts and Nevis | 0.01 (0 - 0.01) | 22.93 (13.04 - 35.27) | 0.01 (0.01 - 0.02) | 17.18 (9.77 - 26.45) | -0.88  (-0.91 - -0.85) |
| Saint Lucia | 0.03 (0.02 - 0.05) | 27.32 (15.59 - 42.09) | 0.03 (0.02 - 0.05) | 17.34 (10.25 - 27.31) | -1.43  (-1.51 - -1.35) |
| Saint Vincent and the Grenadines | 0.03 (0.01 - 0.04) | 25 (14.76 - 39) | 0.02 (0.01 - 0.03) | 18.76 (11.09 - 28.83) | -0.84  (-0.91 - -0.77) |
| Slovak Republic | 1.35 (0.8 - 2.07) | 25.04 (14.69 - 38.72) | 1.16 (0.69 - 1.84) | 21.47 (12.63 - 33.75) | -0.31  (-0.46 - -0.15) |
| Socialist Republic of Viet Nam | 22.01 (13.09 - 34.66) | 35.25 (21.54 - 55.95) | 28.93 (17.29 - 45.79) | 26.33 (15.68 - 41.16) | -0.76  (-0.83 - -0.69) |
| Solomon Islands | 0.22 (0.12 - 0.33) | 79.58 (45.36 - 123.36) | 0.33 (0.19 - 0.51) | 49.81 (28.63 - 78.6) | -1.52  (-1.54 - -1.51) |
| State of Eritrea | 1.15 (0.65 - 1.84) | 40.2 (22.97 - 63.04) | 1.46 (0.84 - 2.31) | 22.01 (12.77 - 34.33) | -1.98  (-2.06 - -1.89) |
| State of Israel | 1.27 (0.7 - 2.05) | 26.46 (14.63 - 42.84) | 2.44 (1.38 - 3.86) | 26.93 (15.26 - 42.66) | -0.09  (-0.16 - -0.02) |
| State of Kuwait | 0.54 (0.31 - 0.86) | 25.02 (14.52 - 39.64) | 1.61 (0.92 - 2.52) | 24.81 (14.48 - 38.07) | -0.3  (-0.41 - -0.19) |
| State of Libya | 1.78 (1.03 - 2.85) | 47.51 (27.15 - 74.9) | 2.12 (1.24 - 3.31) | 25.18 (14.6 - 39.03) | -1.94  (-2.17 - -1.71) |
| State of Qatar | 0.15 (0.08 - 0.24) | 25.08 (14.23 - 38.92) | 0.71 (0.41 - 1.11) | 15.17 (8.8 - 23.74) | -2.68  (-3.13 - -2.24) |
| Sultanate of Oman | 0.75 (0.42 - 1.19) | 37.85 (21.64 - 59.2) | 1.19 (0.68 - 1.87) | 19.31 (11.22 - 30.35) | -2.14  (-2.2 - -2.07) |
| Swiss Confederation | 1.91 (1.09 - 3.03) | 25.39 (14.41 - 39.85) | 2.03 (1.14 - 3.25) | 23.83 (13.2 - 38.23) | -0.08  (-0.2 - 0.05) |
| Syrian Arab Republic | 5.96 (3.48 - 9.35) | 57.02 (33.32 - 87.7) | 4.4 (2.59 - 6.8) | 34.69 (19.99 - 54.59) | -1.68  (-1.92 - -1.44) |
| Taiwan (Province of China) | 11.14 (6.34 - 17.83) | 49.91 (28.49 - 79.86) | 11.62 (7.23 - 17.41) | 43.31 (27.08 - 65.04) | -0.4  (-0.68 - -0.12) |
| Togolese Republic | 1.41 (0.8 - 2.21) | 46.87 (26.85 - 72.71) | 2.22 (1.25 - 3.42) | 27.65 (15.51 - 42.35) | -1.48  (-1.58 - -1.38) |
| Tokelau | 0 (0 - 0) | 110.97 (78.12 - 147.83) | 0 (0 - 0) | 75.2 (52.19 - 102.41) | -1.29  (-1.37 - -1.2) |
| Turkmenistan | 2.42 (1.61 - 3.43) | 63.65 (43.76 - 87.11) | 2.44 (1.69 - 3.25) | 44.92 (31.07 - 59.78) | -0.86  (-1.06 - -0.66) |
| Tuvalu | 0.01 (0.01 - 0.01) | 88.37 (62.66 - 121.5) | 0.01 (0.01 - 0.01) | 57.87 (41.11 - 78.64) | -1.35  (-1.4 - -1.31) |
| Ukraine | 34.34 (24.15 - 46.6) | 69.75 (49.64 - 95.25) | 24 (16.88 - 32.23) | 64.15 (44.86 - 86.85) | 0.02  (-0.13 - 0.17) |
| Union of the Comoros | 0.3 (0.21 - 0.43) | 68.81 (47.66 - 94.12) | 0.29 (0.2 - 0.41) | 35.94 (24.83 - 49.82) | -2.07  (-2.1 - -2.05) |
| United Arab Emirates | 1 (0.66 - 1.41) | 45.26 (30.32 - 64.91) | 2.85 (1.94 - 3.98) | 32.06 (21.68 - 45.79) | -1.52  (-2.03 - -1) |
| United Kingdom of Great Britain and Northern Ireland | 23.21 (15.91 - 31.71) | 41.11 (28.12 - 55.99) | 21.79 (15.22 - 29.21) | 38.44 (27.16 - 52.26) | -0.13  (-0.21 - -0.05) |
| United Mexican States | 47.45 (32.45 - 66.84) | 52.27 (36.22 - 70.6) | 47.28 (32.73 - 64.14) | 34.21 (23.59 - 46.54) | -1.32  (-1.41 - -1.23) |
| United Republic of Tanzania | 18.39 (12.45 - 26.23) | 74.88 (51.61 - 102.04) | 29.68 (20.18 - 41.9) | 49.31 (33.9 - 67.81) | -1.25  (-1.3 - -1.19) |
| United States of America | 122.64 (81.15 - 173.13) | 44.11 (29.64 - 62.51) | 81.02 (58.32 - 105.53) | 26.52 (18.95 - 34.77) | -2.14  (-2.37 - -1.91) |
| United States Virgin Islands | 0.05 (0.04 - 0.07) | 45.89 (32.32 - 63.36) | 0.02 (0.02 - 0.03) | 33.89 (23.55 - 46.47) | -0.98  (-1.06 - -0.91) |

DALYs, disability-adjusted life years; ASDR, age standardized DALY rate; CI, confidence interval; EAPC, estimated annual percentage change; UI, uncertainty interval.

**Supplemental Table 3. ICD-10 codes used in endometriosis**

| **Cause** | **ICD-10 code** |
| --- | --- |
| Endometriosis | N80-N80.9 |

**Supplemental Appendix 1. The details of GBD data collection in endometriosis**

A systematic review of endometriosis prevalence was conducted for GBD 2010. The review consisted of a PubMed search and a systematic review of endometriosis throughout the world. Ovid MEDLINE, EMBASE, CINAHL, CAB abstracts, WHOLIS, and ISGLE database were searched. The search strings for PubMed and EMBASE were as follows:

- PUBMED: (“Endometriosis”[Mesh] OR Endometriosis OR Endometrioses OR Endometrioma OR Endometriomas OR Adenomyosis) AND (“Incidence”[Mesh] OR Incidence OR Incidences OR “Prevalence”[Mesh] OR Prevalence OR Prevalences)
- EMBASE: (‘endometriosis’/exp OR endometriosis OR endometrioses OR endometrioma OR endometriomas OR adenomyosis) AND (‘incidence’/exp OR incidence OR incidences OR ‘prevalence’/exp OR prevalence OR prevalences)

Exclusion criteria for the initial systematic review were reviews, studies that did not provide primary data on epidemiological parameters (eg, commentary), and clearly non-representative studies (eg, of only high-risk pregnant women).

**Supplemental Appendix 2.GATHER Checklist**


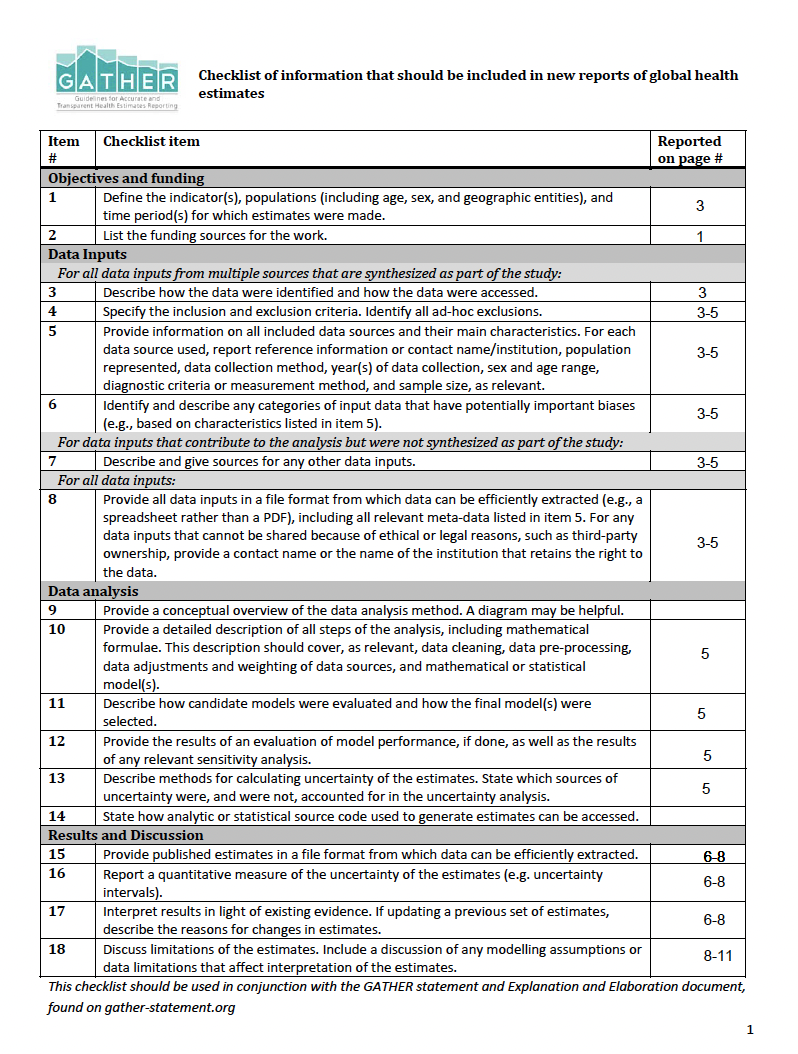

Supplement: Supplementary file 1 [file Data_Sheet_1.docx]
